# Formulation-dependent differences in paclitaxel distribution to anatomical sites relevant to chemotherapy-induced peripheral neuropathy

**DOI:** 10.3389/fphar.2024.1486686

**Published:** 2024-11-06

**Authors:** Milda Girdenytė, Yang Hu, Aghavni Ginosyan, Margareta Hammarlund-Udenaes, Irena Loryan

**Affiliations:** ^1^ Translational Pharmacokinetics/Pharmacodynamics Group (tPKPD), Department of Pharmacy, Faculty of Pharmacy, Uppsala University, Uppsala, Sweden; ^2^ Pharmacy and Pharmacology Center, Institute of Biomedical Sciences, Faculty of Medicine, Vilnius University, Vilnius, Lithuania

**Keywords:** CreEL-paclitaxel, nab-paclitaxel, micellar-paclitaxel, chemotherapy-induced peripheral neuropathy (CIPN), blood-brain barrier, blood-dorsal root ganglion barrier, blood-nerve barrier

## Abstract

**Introduction:**

Chemotherapy-induced peripheral neuropathy (CIPN) is a dose-limiting adverse event observed in patients receiving paclitaxel, associated with initial pathological changes in the peripheral nervous system, i.e., distal nerves and dorsal root ganglia (DRG). The prevalence of CIPN in patients receiving paclitaxel formulated i) in polyethylated castor oil with ethanol (CreEL-PTX), ii) as albumin-bound (nab-PTX), and iii) in XR17 micelles (micellar-PTX), is unexpectedly varying. We hypothesize that the discrepancy in CIPN prevalence could be governed by differences in the extent of paclitaxel distribution across blood-to-tissue barriers at the CIPN-sites, caused by the specific formulation.

**Methods:**

The recently developed Combinatory Mapping Approach for CIPN was used to determine the unbound tissue-to-plasma concentration ratio K_p,uu,tissue_, after a 4-h infusion of 4 mg/kg CreEL-PTX, 4 mg/kg nab-PTX or 1 mg/kg micellar-PTX in male and female Sprague Dawley rats. K_p,uu,tissue_ was determined in conventional (DRG, sciatic nerve) and non-conventional (brain, spinal cord, skeletal muscle) CIPN-sites.

**Results:**

Based on our data, the Cremophor-free paclitaxel formulations were associated with a higher distribution of paclitaxel to CIPN-sites than CreEL-PTX, e.g., K_p,uu,DRG_ of 0.70 and 0.60 for nab-PTX and micellar-PTX, respectively, in comparison to 0.27 for CreEL-PTX (*p* < 0.01). In addition, the fraction of unbound paclitaxel in plasma was on average 1.6-fold higher in nab- and micellar PTX arms and equal to 0.061 and 0.065, respectively, compared to 0.039 for the CreEL-PTX treatment arm (*p* < 0.0001).

**Discussion:**

In the case of similar unbound paclitaxel concentration in the plasma of patients and assumed species-independent extent of paclitaxel transport across the barriers, nab- and micellar-PTX formulations can lead to higher paclitaxel exposure at CIPN-sites in comparison to CreEL-PTX.

## 1 Introduction

Paclitaxel is a microtubule-targeting agent (MTA), widely used in mono- and polytherapy in the treatment of various types of cancer, including breast cancer (Delaloge et al., 2016; Karam et al., 2017; Tan et al., 2019; Paksoy et al., 2023). Although paclitaxel is an effective anticancer drug, its use is limited by several factors, including the development of chemotherapy-induced peripheral neuropathy (CIPN). This is a highly prevalent treatment-limiting adverse event with more than two-thirds of patients experiencing mild to severe CIPN-related symptoms (Seretny et al., 2014). These symptoms are associated with the alteration of neuronal functionality which manifests as paresthesia, hypoesthesia, neuropathic pain, and myalgia in body extremities (Was et al., 2022).

Peripheral nervous system (PNS) tissues, i.e., nerves and dorsal root ganglia (DRG), are the key anatomical sites affected during CIPN development (Peters et al., 2007), herein named conventional CIPN-sites. In addition to PNS, central nervous system (CNS) tissues, brain, and spinal cord, have been discussed as possible non-conventional CIPN-sites both directly and indirectly affected by chemotherapy (Omran et al., 2021). Moreover, Hu et al. observed time-dependent paclitaxel accumulation in skeletal muscle (Hu et al., 2024), a non-conventional CIPN-site, clinically associated with the appearance of myalgia during CIPN development (Was et al., 2022). It is hypothesized, that high unbound paclitaxel exposure at CIPN-sites predisposes CIPN development and is driven by systemic unbound paclitaxel exposure and its transport across the barriers, protecting CIPN-sites (Hu et al., 2024). These barriers include the blood-brain (BBB), blood-spinal cord (BSCB), blood-nerve (BNB), blood-dorsal root ganglion (BDB) barriers, and blood-skeletal muscle interface (BSMI), as well as secondary parenchymal cellular barriers. Differences in CreEL-PTX distribution to different CIPN-sites have been identified in a preclinical model (Hu et al., 2024). It was shown that paclitaxel is actively effluxed at the BBB and BSCB, but is taken up at the BDB, BNB, and BSMI. On the contrary, substantial accumulation inside the cells in the brain parenchyma was observed compared to the DRG parenchymal cells with unbound intracellular-to-extracellular ratio being 11 in the brain and 1.4 in the DRG (Hu et al., 2024). In addition to a generally high risk of CIPN development associated with paclitaxel treatment, a discrepancy in CIPN prevalence between patients receiving different paclitaxel formulations has been observed (Liu et al., 2021; Borgå et al., 2019a), which we hypothesize to be due to differences in tissue distribution attributed to the formulations.

The high lipophilicity of paclitaxel has led to the development of different formulations with enhanced paclitaxel solubility. These formulations (Table 1) include paclitaxel solubilized in a mixture of Cremophor EL and ethanol (50:50, v:v, CreEL-PTX), albumin-bound nanoparticles (nab-PTX), and a surfactant XR17, derived from retinoic acid (micellar-PTX). The first approved paclitaxel formulation, CreEL-PTX, contains a vehicle that has been associated with hyperlipidemia, neurotoxicity, and most commonly, hypersensitivity reactions (Gelderblom et al., 2001). Due to these reactions, CreEL-PTX is administered at a lower dose, with an extended infusion time after premedication, which includes H1-antagonists, H2-blockers, or corticosteroids like dexamethasone (Dubinsky et al., 2022). Dexamethasone remains the main premedication drug in chemotherapy (Barroso et al., 2024). Novel Cremophor-free formulations include nab-PTX and micellar-PTX, which differ from CreEL-PTX in their dosing regimens and systemic pharmacokinetic properties (Table 1). With these new vehicles, the use of paclitaxel has been extended enabling the use of higher dosing regimens. Yet, CIPN remains a prominent dose-limiting adverse event, with varying CIPN prevalence and severity depending on the paclitaxel formulation. Distinct CIPN prevalence has been described for the different paclitaxel formulations. The risk of CIPN development for CreEL-PTX has been associated with a longer time above total paclitaxel concentrations in plasma of 0.05 μmol/L (T_>0.05,_ equivalent to 42.7 ng/mL) and overall systemic paclitaxel exposure, calculated by multiplying area under the curve from time 0 to infinity (AUC_∞_) by weeks of therapy (Mielke et al., 2005). Meta-analysis showed an overall higher CIPN prevalence in breast cancer patients receiving nab-PTX treatment in comparison to CreEL-PTX in a range of different dosing regimens with odds ratio (OR) of 2.10, 95%CI: 1.37–3.23 (*p* = 0.001) in any grade and OR of 4.01, 95%CI: 2.51–6.41 (*p* < 0.001) in grade ≥3 of neuropathy (Liu et al., 2021). Micellar-PTX showed a similar incidence rate of CIPN compared to CreEL-PTX (Vergote et al., 2020). In contrast, a randomized cross-over study comparing nab-PTX and micellar-PTX with the same dosing regimen identified a two-fold higher incidence of CIPN-related symptoms in the micellar-PTX arm, such as paresthesia and pain in extremities (Borgå et al., 2019a). To our knowledge, no study has compared all three formulations on their distribution to the nervous system sites, relevant to CIPN development, which leads to an insufficient understanding of the formulation’s impact on CIPN development.

**TABLE 1:** Comparison between CreEL-PTX, nab-PTX and micellar-PTX regarding their formulation, year of approval, dosing regimens and systemic pharmacokinetic parameters in humans after the most common dosing regimen.

Formulation	CreEL-PTX (Sparreboom et al., 2005)	nab-PTX (Sparreboom et al., 2005)	micellar-PTX (Borgå et al., 2019b)
	paclitaxel dissolved in Cremophor EL/ethanol	albumin-bound, nanoparticle paclitaxel	paclitaxel with surfactant XR17
Approval year	1992 (FDA^a^) 1993 (EMA^a^)	2005 (FDA) 2008 (EMA)	2018 (EMA)^b^
The most often used dose (mg/m^2^)	175	260	250
Most often used duration of IV^c^ infusion (hours)	3	0.5	1
Mean area under the total plasma concentration-time curve, AUC_∞_ (ng/h/mL)^d^	12 603	14 789	15 985
Min – max values of clearance, CL (L/h/m^2^)	10.2–28.8	8.7–43.4	4.4–22.6
Min – max values of apparent volume of distribution at steady-state, V_ss_ (L/m^2^)	99.7–346.0	53.2–492.9	23.8–165.0
Mean fraction of unbound paclitaxel in human plasma, f_u,plasma_	0.023	0.062	0.053
Estimated mean area under the unbound plasma concentration-time curve, AUC_∞_ (ng/h/mL)^d^	289.9	916.9	847.2

^a^ EMA, European Medicine Agency; FDA, Food and Drug Administration.

^b^ According to the EMA, statement; micellar-PTX, is withdrawn from the market on the 9^th^ of February 2024 (EMA, 2024).

^c^ IV, intravenous infusion.

^d^ AUC_∞_ - area under the curve from time 0 to infinity.

We hypothesize, that in addition to the existing differences in the extent of paclitaxel transport across the different barriers, governed by structural and functional dissimilarities between them, paclitaxel formulations and premedication may also influence the extent of paclitaxel’s transport to CIPN-sites. This was illustrated by Li et al., who showed distinct total paclitaxel tissue distribution between paclitaxel formulations in mice, including CreEL-, nab-, and micellar-PTX (Li et al., 2018). In the case of premedication, experiments in rats showed that dexamethasone can affect the BBB by restoring its integrity and modulating the endothelial barrier permeability after inflammation-induced tissue damage *in vivo* (McMahon et al., 2020). Additionally, dexamethasone can induce paclitaxel metabolism and facilitate its systemic elimination (Anderson et al., 1995). This could be a potential explanation for decreasing paclitaxel’s anti-tumor effect as shown in mice (Pang et al., 2006).

Differences in the extent of distribution should be investigated in male and female animals, however, this variability is unexplored in the case of paclitaxel. Two behavioral studies in rats and mice observed contradictory results related to CIPN development after paclitaxel administration (Hwang et al., 2012; Naji-Esfahani et al., 2016), but possible differences in the extent of distribution are unknown. This emphasizes the need to include animals of both sexes in the study and evaluate the potential sex differences in paclitaxel transport to CIPN-sites, focusing on comparing paclitaxel formulations in paclitaxel distribution to the nervous system.

Key methodological aspects need to be considered when evaluating the extent of distribution to the tissues of interest. Generally, the total tissue-to-plasma concentration ratio, K_p,tissue_, has been evaluated using respective AUCs (Leblanc et al., 2018; Nakamura et al., 2017). However, to determine the extent of transport across tissue membranes, the unbound partition coefficient, K_p,uu,tissue_, needs to be assessed, as only unbound molecules are pharmacologically/toxicologically active and are transported across barriers (Hammarlund-Udenaes et al., 2008; Summerfield et al., 2006). To evaluate this parameter, the Combinatory Mapping Approach for CIPN (CMA-CIPN), can be used (Hu et al., 2024). This includes obtaining K_p,tissue_ values and correcting them for plasma protein binding and tissue distribution and binding to determine K_p,uu,tissue_ (Hu et al., 2024; Loryan et al., 2014).

The overarching goal of this study was to systematically compare the distribution of paclitaxel to CIPN-sites when administered as CreEL-PTX, nab-PTX, and micellar-PTX. This comparison aimed to identify pharmacokinetic factors potentially contributing to differences in CIPN development observed across these formulations. The CMA-CIPN methodology (Hu et al., 2024) was used for the assessment of the extent of paclitaxel transport across the BBB, BSCB, BNB, BDB, and BSMI to CIPN-sites in male and female rats. Additionally, the impact of dexamethasone premedication on CreEL-PTX distribution to CIPN-sites was explored. Acquired data shows that the extent of paclitaxel distribution into the conventional CIPN-site DRG is at least 2-fold higher when administered in the form of nab-PTX and micellar-PTX in comparison to CreEL-PTX. Conversely, dexamethasone premedication had no impact on the extent of paclitaxel distribution after CreEL-PTX administration. The findings provide new insights into how clinically used paclitaxel formulations may affect the paclitaxel transport to CIPN-sites which may consequently be reflected in CIPN manifestations.

## 2 Methods

To examine how paclitaxel formulations may differ in the extent of unbound paclitaxel distribution to CIPN-sites, the CMA-CIPN approach, combining *in vivo* and *in vitro* elements, was used (Hu et al., 2024). *In vivo* pharmacokinetic experiments were performed in rats of both sexes, receiving intravenous (IV) infusions of one of the paclitaxel formulations. Additionally, within the CreEL-PTX treatment arm, animals received paclitaxel formulation with or without dexamethasone premedication (Table 2). Further, equilibrium dialysis was performed to evaluate the fraction of unbound paclitaxel in plasma using terminal plasma samples from animals receiving different paclitaxel formulations. The collected data was used to quantitatively examine the extent of unbound paclitaxel transport across CNS, PNS barriers, and skeletal muscle interface, measured using K_p,uu,tissue_.

**TABLE 2:** Treatment arms included in the *in vivo* experiments with the number and sex of animals, estimated total, loading, and maintenance doses.

Treatment arm	Animals included	Estimated total dose (mg/kg)	Estimated loading dose^a^ (mg/kg)	Estimated maintenance dose^b^ (mg/kg)
CreEL-PTX alone (IV)	5M/3F	4.4	3.17	1.28
CreEL-PTX (IV) + 0.15 mg/kg dexamethasone (IM)	4M	4.4	3.17	1.28
CreEL-PTX (IV) + 0.30 mg/kg dexamethasone (IM)	4M	4.4	3.17	1.28
nab-PTX (IV)	3M/4F	3.9	3.17	0.76
Micellar-PTX (IV)	4M/2F	3.9	3.17	0.76

^a^ The rate of infusion of the loading dose was 0.053 mL/min for all treatment arms, while

^b^ the rate of infusion of maintenance dose was 0.003 mL/min for CreEL-PTX, arms and 0.002 mL/min for nab- and micellar-PTX, arms.

Abbr. IM, intramuscular; IV, intravenous; F, female; M, male.

### 2.1 Materials

Paclitaxel 6 mg/mL injection solution in Cremophor EL/Ethanol 1:1, v:v (Paclitaxel Actavis, Teva Sweden AB), nanoparticle albumin-bound powder containing 100 mg of paclitaxel (Abraxane, Celgene AB), 60 mg powder of micellar paclitaxel (Apealea, Inceptua AB), 4 mg/mL solution of dexamethasone phosphate (Dexacur, Abbroxia AB), heparin 5000 IU/mL solution for injection (Heparin LEO, LEO Pharma AB), sodium chloride solution for injection 9 mg/mL and isoflurane liquid for inhalation (IsoFlo vet, Zoetis Animal Health ApS) were all obtained from Apoteket Production & Laboratories AB (Stockholm, Sweden). Paclitaxel-d5 and paclitaxel (HPLC-grade) were purchased from Toronto Research Chemicals (Toronto, Canada). Dimethyl sulfoxide (DMSO) was purchased from MP Biomedicals (Eschwege, Germany) and formic acid was obtained from Sigma-Aldrich (Stockholm, Sweden). Acetonitrile (ACN) was obtained from Supelco (Darmstadt, Germany). All chemicals and reagents used in experiments were of analytical grade. The water used was deionized in-house and purified with a Milli-Q Academic system (Millipore, Bedford, MA, United States; Resistance 18.2 Ω; Millipak®Express 20 Filter, 0.22 μm) was purchased from Merck Millipore (Burlington, MA, United States).

### 2.2 Animals

Twenty-six male and 9 female Sprague-Dawley rats were used for the *in vivo* and *in vitro* experiments (Taconic, Lille Skensved, Denmark). A sample size of at least 3 individuals per arm was calculated to attain a significance level of 0.05 and power of 80% to identify a 2-fold difference in total and unbound paclitaxel tissue-to-plasma ratios between treatment arms. Due to a lack of prior information on the variability of evaluated parameters in the nab- and micellar-PTX arms, similar variability in the same parameter within the arms was assumed and later confirmed in the experiments.

All animals were 8–12 weeks old and weighed 250–300 g during the experiments. After arrival, the animals were housed in groups under temperature- and humidity-controlled conditions, 20°C–22°C and 40%–50% humidity, in a 12-h light/dark cycle with *ad libitum* food and water. All animals were acclimatized for 1 week before the experiment. Experiments were performed following guidelines from the Swedish National Board for Laboratory Animals, approved by the Animal Ethics Committee of Uppsala, Sweden (Ethical Approval Dnr 5.8.18-12230/2019). Performed studies were not randomized or blinded for the treatment arms. The data reporting follows the ARRIVE guidelines (Percie du Sert et al., 2020).

### 2.3 *In vivo* pharmacokinetic studies

To determine the difference in the extent of paclitaxel distribution between the clinically used formulations, quantification of the extent of total paclitaxel distribution to CIPN-sites, characterized by total tissue-to-plasma concentration ratio, K_p,tissue_, is needed. Thus, *in vivo* pharmacokinetic studies were performed to compare K_p,tissue_ values between different paclitaxel formulations. This was determined at the end of a 4-h IV infusion. In addition, to examine the impact of dexamethasone on the extent of paclitaxel distribution to CIPN-sites, dexamethasone was administered as an intramuscular injection (IM) in the CreEL-PTX arm 30 min before CreEL-PTX administration. Details of the treatment arms and dosing regimen are provided in Table 2.

#### 2.3.1 Dosing regimen selection

To minimize the use of animals and increase our knowledge of inter-individual variability in pharmacokinetic parameters, K_p,tissue_ was determined at a pseudo-steady-state using a terminal single time point (Hammarlund-Udenaes et al., 2008). It is important to bear in mind that it is necessary to reach steady-state in plasma and tissues to accurately determine K_p,tissue_. In the case of paclitaxel, the steady-state in plasma could be achieved via 4-h IV infusion, yet, the time to steady-state in tissues requires up to 240 h of infusion (Hu et al., 2024). Nevertheless, in this study, 4-h IV infusions were used to determine K_p,tissue_.

According to the free-drug theory (Summerfield et al., 2022), only unbound drug is able to cross membranes. Hence, it is assumed that the drug needs to be dissociated from its vehicle before passing through the endothelial barriers. Therefore, after paclitaxel dissociation from its vehicle and the passage across the endothelial cells, the intra-tissue distribution of paclitaxel, i.e., from the interstitial fluid to the cells, is independent of its formulation. Consequently, the only difference between formulations in K_p,tissue_ is linked to plasma protein binding and the net flux of paclitaxel across investigated barriers, as it is determined at the same time point for all formulations. Hence, targeting the pseudo-steady-state, i.e., steady-state in plasma, but not tissue concentrations, achieved after the 4-h IV infusion is evaluated to be a valid assumption when comparing paclitaxel’s distribution after administration of the different formulations.

To reach a steady-state in plasma faster, the infusion was administered as a combination of a loading dose, administered for 30 min, followed by a maintenance dose given during 210 min. The loading and maintenance doses were calculated using Equation 1 and Equation 2, respectively, where C_tot,plasma,ss_ is the targeted plasma concentration (mg/L) at the steady-state, V_d_ is the volume of distribution (L/kg) and CL is the elimination clearance (L/h/kg).

$$\text{Loading dose}=C_{\text{tot},\text{plasma},\text{ss}}\times V_{d}$$ (1)

$$\text{Maintenance dose}=C_{\text{tot},\text{plasma},\text{ss}}\times \text{CL}$$ (2)

In the ideal situation, experimental conditions between the treatment arms should be selected. Therefore, the same C_tot,plasma,ss_ should be targeted. Herein, we have arbitrarily selected C_tot,plasma,ss_ of ca 170 ng/mL, which is commonly reached in clinical settings after administration of CreEL-, nab-, and micellar-PTX in humans. The selection was based on multiple grounds and supported by available clinical data. According to Sparreboom et al. (2005) after CreEL-PTX (175 mg/m^2^ over 3 h) and nab-PTX (260 mg/m^2^ over 30 min) administration, paclitaxel exposure levels were similar between patients’ groups after 10 h and ranging from 100 to 250 ng/mL. In the case of micellar-PTX, Borgå et al. (2019a) showed similar paclitaxel exposure levels compared to nab-PTX after 260 mg/m^2^ over 1 h with a range from 100 to 250 ng/mL. In addition, given the expected low concentrations in the brain and spinal cord, the selected concentration was sufficiently high to quantify total paclitaxel concentration in all tissues of interest.

The loading and maintenance doses of CreEL-PTX were estimated by using a volume of distribution (V_d_) of 18.3 L/kg and clearance (CL) of 1.9 L/h*kg, determined in rats (Hu et al., 2024). In the case of nab-PTX, the doses were estimated by using pharmacokinetic data from the literature, with V_d_ of 18.3 L/kg and CL of 1.1 L/h*kg estimated in rats (Sparreboom et al., 2005). A clinical trial found no systemic pharmacokinetic differences between micellar-PTX and nab-PTX (Borgå et al., 2019a). Therefore, the same dosing regimen as nab-PTX was chosen for micellar-PTX. This resulted in an overall similar estimated total paclitaxel dose in all treatment arms (Table 2).

The estimated loading and maintenance doses were used to calculate the rate of infusion (mL/min), Equation 3, where BW is the weight of the rat (kg), $C_{infusion\;solution}$ is the paclitaxel concentration in the prepared infusion solution (mg/mL) and time of infusion is expressed in minutes.

$$\text{Rate of infusion}=\frac{Dose\times BW}{C_{infusion\;solution}\times Time\;of\;infusion}$$ (3)

A concentration of 0.6 mg/mL of paclitaxel infusion solutions in saline was selected and prepared on the day of the experiment following the manufacturer’s instructions. The CreEL-PTX injection solution of 6 mg/mL was diluted in saline and vortexed for 1 min before use. In the case of nab- and micellar-PTX, the required powder amount was taken and wetted with saline for 5 min. The solutions were slowly swirled to avoid foaming. To control the paclitaxel solutions and their stability during the experiment, samples from the syringes were taken at 0 and 4 h.

To investigate the premedication effect on the extent of paclitaxel distribution after different dexamethasone dose administration, dexamethasone doses of 0.15 mg/kg and 0.3 mg/kg were selected, to reach similar dexamethasone exposure in plasma in rats as it is reached in humans after IV bolus injection of 10 and 20 mg of dexamethasone, respectively (Bashir and Acosta, 2020). The selected doses of dexamethasone were injected IM in the right hind leg. The selection of the administration route was based on previous studies showing similar dexamethasone systemic pharmacokinetic profiles after IM and IV administration in rats (Samtani and Jusko, 2005). The injection solution of dexamethasone was prepared by diluting the dexamethasone solution with saline to 0.5 mg/mL, resulting in a volume injected IM of less than 0.2 mL.

#### 2.3.2 Experimental procedure

The day before the experiment, surgical implantation of polyethylene (PE) catheters to the femoral artery and vein was performed under anesthesia, by inhalation of 2.5% is oflurance balanced with 3 L/min oxygen. After surgery, rats were individually placed into a CMA120 system for freely moving animals (CMA, Solna, Sweden) with *ad libitum* access to food and water for 24 h. The paclitaxel infusion and blood sampling were performed the next day. The paclitaxel solution was administered via the venous catheter using a Syringe Infusion Pump 22 (Harvard apparatus, Massachusetts, United States).

Blood samples (∼180 µL) were collected from the arterial catheter at 0, 1, 2, 3, and 4 h after the start of paclitaxel infusion. All samples were taken into heparinized Eppendorf tubes (25 IU of heparin per tube, 5 µL). Samples were immediately centrifuged at 10 000 rpm for 5 min at room temperature. After centrifugation, plasma was collected and stored at −20°C until the bioanalysis.

At the end of each experiment, the rats were anesthetized by inhalation of 2.5% is oflurance balanced with 3 L/min oxygen. To minimize residual blood in tissues of interest, terminal blood was withdrawn via cardiac puncture into a 6 mL heparin-containing vacutainer (Mediq, Kungsbacka, Sweden). Terminal tissue sampling was performed after decapitation. Brain (Br), sciatic nerve from both sides (SN), skeletal muscle (*biceps femoris*, SM), cervical and thoracic parts of the spinal cord (SC), and DRG from the lumbar region were collected. The brain was wrapped in pre-weighed aluminum foil. SC and SM were collected in pre-weighed 2 mL pre-filled bead tubes (VWR^®^ Hard Tissue Homogenizing Mix, 2.8 mm Ceramic Beads; Stockholm, Sweden), while SN and DRG were collected in pre-weighed 0.5 mL pre-filled bead tubes (VWR^®^ Soft Tissue Homogenizing Mix, 1.4 mm Ceramic Beads; VWR, Stockholm, Sweden). All samples were weighed and kept on dry ice until the end of the sample collection. In the experiments with dexamethasone premedication, SM was collected from the other side than the dexamethasone injection site. After collecting all the samples, they were stored at −20°C pending bioanalysis.

### 2.4 Plasma protein binding assay

To evaluate the fraction of unbound paclitaxel in plasma, f_u,plasma_, in the different treatment arms, equilibrium dialysis was performed according to the method by Kalvass and Maurer (2002), with modifications. Plasma samples, received from heart puncture from 8 drug-naive rats (N = 8) and selected representative samples from the *in vivo* experiments from 16 rats (N = 16) were used for the equilibrium dialysis (Table 3).

**TABLE 3:** Plasma protein binding of paclitaxel determined in various treatment arms. Data presented as a mean ± SD.

Type of treatment	Sex of animal	Number of biological (N) and technical (n) replicates	f_u,plasma_
Spiked naïve rat plasma with vehicle-free PTX	male	N = 8, n = 2–8	0.069 ± 0.006
4-h IV infusion of CreEL-PTX alone	male	N = 1, n = 2	0.039 ± 0.003
	female	N = 2, n = 2	
4-h IV infusion of CreEL-PTX with 0.15 mg/kg dexamethasone premedication	male	N = 3, n = 2	0.036 ± 0.007
4-h IV infusion of CreEL-PTX with 0.30 mg/kg dexamethasone premedication	male	N = 2, n = 2	0.044 ± 0.004
4-h IV infusion of nab-PTX	male	N = 2, n = 2	0.061 ± 0.009
	female	N = 2, n = 2	
4-h IV infusion of micellar-PTX	male	N = 2, n = 2	0.065 ± 0.012
	female	N = 2, n = 2	

Abbreviations: IV, intravenous.

A Teflon 96-well plate HTD96b fitted with a regenerated cellulose membrane (molecular weight cutoff 12–14 kDa) was used (HTDialysis LLC, Gales Ferry, CT, United States). The day before the equilibrium dialysis, the cellulose membrane was treated according to the recommendations from the manufacturer. On the day of the experiment, the plasma samples were thawed and pH was measured and adjusted to 7.4, if needed.

Fifty µg/mL paclitaxel stock in acetonitrile (containing the pure drug without any formulation) was used to obtain a final paclitaxel concentration of 1,000 nM (854 ng/mL) in Eppendorf tubes. The selection of 1,000 nM was decided to have concentrations higher than the limit of quantification of paclitaxel in the buffer side at the end of the equilibration period, as paclitaxel is highly protein-bound. Acetonitrile was evaporated under nitrogen before spiking with blank plasma followed by vortexing for 1 min. One hundred µL of spiked plasma or 100 µL of plasma from *in vivo* experiments was dialyzed against an equal volume of phosphate-buffered saline (PBS), pH 7.4 for 6 h at 37°C and 200 rpm in an incubator with orbital shaking (MaxQ4450 Thermo Fisher Scientific, NinoLab, Sweden). To evaluate drug recovery and thermostability in plasma during the experiment, 50 µL of spiked plasma samples were taken before and after the 6 h incubation, mixed with 50 µL PBS, and stored at −20°C pending bioanalysis.

At the end of the incubation, 50 µL of buffer (receiver side) was sampled and subsequently mixed with 50 µL of blank plasma, and 50 µL of plasma (donor side) was sampled and subsequently mixed with 50 µL of PBS. All samples were collected in 96-well plates.

The unbound fraction of paclitaxel in plasma, f_u,plasma_ was calculated with Equation 4.

$$f_{u,\text{plasma}}=\frac{C_{buffer}}{C_{plasma}}$$ (4)

The spiking recovery was calculated according to Equation 5 to examine if the initial concentration of paclitaxel was similar to the theoretical level. C_0h_ and C_theoretical_ are the measured and theoretical concentrations in the plasma before the incubation (at 0 h), respectively.

$$\text{Spiking recovery }\left(\%\right)=\frac{C_{0h}}{C_{\text{theoretical}}\;}\times 100\%$$ (5)

The thermostability of paclitaxel was calculated with Equation 6. A range of 100% ± 30% was considered acceptable (Di et al., 2012). C_0h_ and C_6h_ are the measured concentrations in spiked plasma before and after 6 h incubation, respectively.

$$\text{Thermostability as }\%\text{remaining}=\frac{C_{6h}}{C_{0h}}\times 100\;\%$$ (6)

### 2.5 Bioanalysis

The samples from *in vivo* and *in vitro* studies were analyzed using previously validated methods (Hu et al., 2024; Balayssac et al., 2023). Bioanalysis was performed using Waters Acquity ultra-performance liquid chromatography (UPLC) coupled with Waters Xevo TQ-S Micro triple quadrupole mass spectrometer (MS/MS) (Waters Corporation, Milford, MA, United States). Data acquisition and quantitation was done using Masslynx v4.2 (Waters Corporation, Milford, MA, United States).

#### 2.5.1 Sample preparation for bioanalysis

Standards (Std) and quality control samples (QC) were prepared in several matrices: undiluted blank rat plasma, 1:1 (v:v) blank plasma in PBS, and 1:4 (w:v) blank brain homogenate in PBS. Calibration ranges were 0.5–2000 ng/mL for undiluted plasma and 0.1–500 ng/g for diluted brain homogenate. Stds prepared in blank brain homogenate were used to determine paclitaxel concentration in all CNS, PNS tissues, and skeletal muscles. The quality control samples for plasma and brain matrices had concentrations of 0.4, 4, 40, and 400 ng/mL. A calibration curve in the range of 1–1,000 nM in 1:1 blank plasma in PBS was used to analyze samples from the equilibrium dialysis experiment.

Tissue samples were homogenized before the sample preparation. The right brain hemisphere was homogenized mechanically in 1:4 (w:v) with PBS, pH 7.4, using a Heidolph mechanical stirrer (Heidolph instruments GmbH & Co., Schwabach, Germany) followed by ultrasonication with ultrasonic processor VCX-130 (Sonics, Chemical Instruments AB, Stockholm, Sweden). SC and SM were diluted 1:4, while PNS tissues were diluted 1:9 due to their smaller weight and expected higher concentrations compared to CNS tissues. All tissues except the brain were homogenized using a 4-Place Beads Homogenizer (VWR, Stockholm, Sweden) at a speed of 5,000 rpm for 1 min, except SM, which was homogenized for 2 min until no pieces of the tissue were seen in the homogenate during visual inspection.

Sample preparation for bioanalysis of plasma and tissue samples, Stds, QCs, and respective blanks was performed in two steps. Fifty µL of a sample was precipitated with 150 μL ACN, containing 50 ng/mL paclitaxel-d5 as internal standard, and was vortexed for 30 s. The samples were then centrifuged for 3 min at 13 000 rpm, followed by diluting 100 µL of the supernatant 1:1 with 0.1% formic acid in water, vortexing for 10 s, and placing the sample into an autosampler set at 4°C. Ten µL of the sample was injected into the column.

#### 2.5.2 UPLC-MS/MS conditions and instrumentation

Paclitaxel and internal standard paclitaxel-d5 were analyzed in positive electrospray ionization (ESI+) mode to obtain protonated parent molecular ions. Quantification was performed using multiple reaction monitoring (MRM) mode to monitor Parent → Product ion m/z transitions 854.20 → 286.00 for paclitaxel and 859.20 → 291.00 for paclitaxel-d5. Nitrogen was used as a nebulizing and desolvation gas with a flow rate of 10 L/h and 800 L/h, respectively. Argon was used as a collision gas. For both compounds 10.0 V (V) cone voltage and 20.0 V collision energy were used with source temperature set at 150°C and desolvation temperature set at 500°C.

A Waters ACQUITY UPLC BEH C18 column, 2.1 × 50 mm, 1.7 μm, protected by an ACQUITY UPLC BEH C18 VanGuard pre-column, 2.1 × 5 mm, 1.7 μm, (Waters Corporation, Milford, MA, United States) was used to separate analytes. Gradient elution of mobile phases, 0.1% formic acid (FA) in MQ-water (MPA), and 0.1% FA in acetonitrile (MPB) were used with a flow rate of 0.3 mL/min. The initial conditions were set as follows: 95% of MPA and 5% of MPB for 2.5 min, changing to 5% of MPA and 95% of MPB from 2.5 to 3.0 min, and returning to the starting concentrations. The total run time was 3.5 min. The retention times for paclitaxel and internal standard paclitaxel-d5 were on average 2.4 min.

The linearity of the calibration curves was tested by linear regression analysis, with the best fit using a weighing function of 1/x^2^. All curves were linear with determination coefficients R^2^ equal to or higher than 0.99. Lower (LLOQ) and upper (ULOQ) limits of quantification were set to the lowest and highest concentration levels in each standard curve, respectively. A matrix-matched external standard and coeluting internal standard method was used. To avoid any potential impact of carryover on the samples, blank samples or MPA were included after ULOQ standards, samples with an expected high concentration, and before the next study sample. The acceptable accuracy of each standard and quality control level concentration was set to be ±15%, except for the low level of quantification, which was set to ±20% of the nominal values according to ICH guidelines (EMA, 2022). The bioanalytical method was validated according to the FDA guidance (FDA, 2022).

### 2.6 Data analysis

The total tissue-to-plasma ratio, K_p,tissue_, was using Equation 7.

$$K_{p,\text{tissue}}=\frac{C_{tot,tissue}}{C_{tot,plasma,ss}}$$ (7)

where C_tot,tissue_ is the total paclitaxel concentration in the tissue, and C_tot,plasma,ss_ is the total paclitaxel concentration in the terminal plasma sample.

The unbound tissue-to-plasma concentration ratio, K_p,uu,tissue_ was calculated using Equation 8. According to the CMA-CIPN approach, K_p,uu,tissue_ calculations are based on K_p,tissue_ values, plasma protein binding and tissue distribution (Hu et al., 2024; Loryan et al., 2014). The error propagation method was used to calculate mean ± standard deviation (SD) (Loryan et al., 2017).

$$K_{p,\text{uu},\text{tissue}}=\frac{K_{p,tissue}}{V_{u,tissue}\;x\;f_{u,plasma}}$$ (8)

where V_u,tissue_ is the unbound volume of distribution of drug in the tissue, describing the active uptake into tissues and paclitaxel distribution inside the tissue. It is governed by active transport, pH partitioning, and specific and nonspecific binding that was previously determined using the novel tissue uptake assay (Hu et al., 2024).

### 2.7 Statistical analysis

Statistical analyses were performed using GraphPad Prism 9.3.1 for Windows (GraphPad Software, San Diego, CA, United States). Normality was tested with the Shapiro-Wilk test. The statistical significance in differences between K_p,tissue_ among treatment arms was determined by a two-way ANOVA test followed by Tukey’s multiple comparisons, with treatment and tissue being two factors. The f_u,plasma,_ and K_p,uu,tissue_ values were analyzed using one-way ANOVA followed by Tukey’s multiple comparisons, and unbound tissue-to-plasma concentration ratios were evaluated separately for each tissue. A *p* < 0.05 was taken as a significant difference. Data are expressed as mean ± standard deviation (SD). The mean and SD of K_p,uu,tissue_ were determined following the law of propagation of error (Loryan et al., 2017).

## 3 Results

### 3.1 Total and unbound paclitaxel exposure in plasma in different treatment arms

**FIGURE 1:**
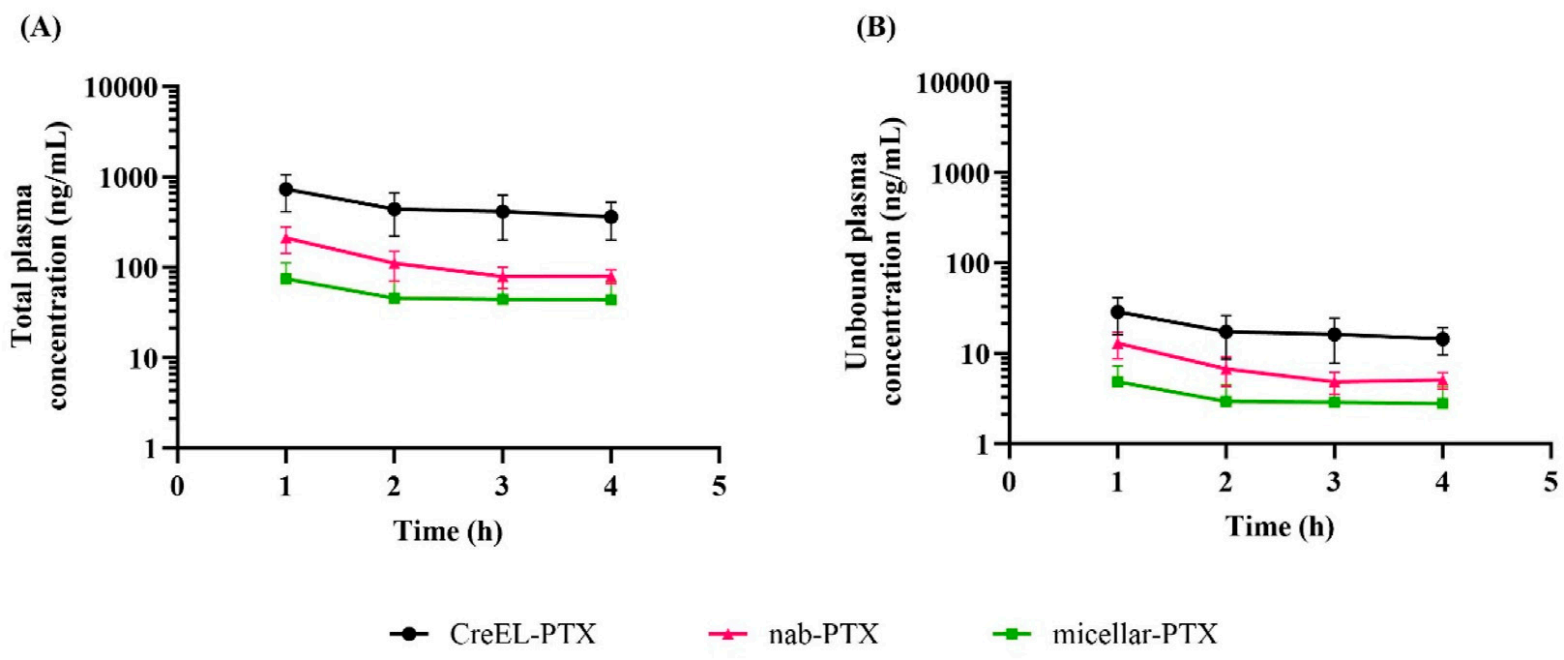
Total and unbound plasma pharmacokinetics of paclitaxel after a 4-h intravenous infusion of 4 mg/kg CreEL-PTX (N = 8, black), 4 mg/kg nab-PTX (N = 7, magenta) and 1 mg/kg micellar-PTX (N = 6, green) formulations. Semilogarithmic plot showing total **(A)** and unbound **(B)** paclitaxel plasma concentrations (ng/mL) over time. Data presented as mean ± SD.

Total plasma concentrations at 4 h after the CreEL-PTX, nab- and micellar-PTX infusion were 369 ± 124 ng/mL, 83 ± 17 ng/mL, and 43 ± 22 ng/mL, respectively (Figure 1A). Due to high protein binding in plasma, the unbound paclitaxel exposure at steady-state was 14.4 ± 4.8 ng/mL, 5.1 ± 1.0 ng/mL, and 2.8 ± 1.5 ng/mL in the groups, respectively (Figure 1B), while the achieved total paclitaxel concentrations in tissues are presented in the Supplementary Material. Data analysis showed pronounced, but not statistically significant differences in plasma exposure between male and female rats. For example, at the end of CreEL-PTX infusion total plasma concentration was 296.6 ± 89.56 ng/mL and 490.1 ± 50.6 ng/mL in male and female rats, accordingly. The difference in total and unbound paclitaxel exposure in plasma between CreEL-, nab- and micellar-PTX was partially due to identified lower paclitaxel concentrations in the 0 and 4 h syringes of micellar-PTX compared to the two other formulations. This was reflected in the mean administered doses of 4 mg/kg in CreEL- and nab-PTX treatment arms, while in the micellar-PTX treatment arm, it was determined to be 1 mg/kg, which is different from estimated doses (Table 2). Although similar exposure levels could not be attained in the different treatment arms in the present study, we could compare the formulations as it was earlier shown that K_p,tissue_ remains unchanged at unbound paclitaxel plasma concentrations between 0.37 and 15.2 ng/mL (Hu et al., 2024). Therefore, further comparison between paclitaxel formulations regarding the extent of paclitaxel distribution was performed.

### 3.2 Paclitaxel formulation changes the fraction of unbound paclitaxel in plasma

The fraction of unbound paclitaxel in plasma was assessed in drug naïve rats (blank, control) and in the terminal plasma samples collected at the end of paclitaxel infusion (Table 3). Due to similar f_u,plasma_ between male and female rats from the same treatment arm (data not shown), results were combined. In the drug naïve rat plasma, mean f_u,plasma_ of vehicle-free paclitaxel was 0.069. This was significantly higher than the mean f_u,plasma_ of 0.039 measured in the CreEL-PTX treatment arm (*p* < 0.0001). However, the mean f_u,plasma_ in nab- and micellar PTX arms were 0.061 and 0.065, respectively, which did not significantly differ from the data from the drug-naïve rat plasma.

No significant difference in f_u,plasma_ was observed between CreEL-PTX alone and after dexamethasone premedication arms with the mean f_u,plasma_ of 0.036 and 0.044 in CreEL-PTX after 0.15 mg/kg and 0.3 mg/kg dexamethasone premedication, respectively. Therefore, all biological and technical replicates from CreEL-PTX with or without premedication arms were included to calculate the mean f_u,plasma_ of paclitaxel after CreEL-PTX administration, which was determined to be 0.039. This parameter was then used to assess K_p,uu,tissue_ in all CreEL-PTX arms.

### 3.3 The extent of unbound paclitaxel distribution across the endothelial barriers varies depending on paclitaxel formulation

To compare the net flux across tissue membranes of interest between formulations, the extent of unbound paclitaxel distribution was determined across CNS, PNS barriers, and BSMI using the CMA-CIPN methodology (Equation 8). The extent of unbound paclitaxel distribution was the lowest across the BBB and BSCB, with mean K_p,uu,Br_ being 0.006 and 0.010 and K_p,uu,SC_ being 0.004 and 0.009 in the CreEL- and nab-PTX arms, respectively (Table 4). More than 10-fold higher K_p,uu,tissue_ values were observed in SN and DRG sites in comparison to CNS (*p* < 0.001), with mean K_p,uu,SN_ being 0.89 and 1.71, and K_p,uu,DRG_ being 0.27 and 0.60 in the CreEL- and micellar-PTX treatment arms, respectively. Higher than unity K_p,uu,SM_ was observed in all treatment arms, being 1.68, 2.98, and 3.30 in the CreEL-, nab- and micellar-PTX arms, respectively. Independent of tissue, the observed K_p,uu,tissue_ in the CreEL-PTX arm was lower than in the two other treatment arms. K_p,uu,tissue_ values were not different between nab- and micellar-PTX arms for the same tissue (Table 4).

**TABLE 4:** K_p,tissue_ and K_p,uu,tissue_ values shown as mean ± SD in the different treatment arms. Details of statistical tests are presented in the Supplementary Material.

Formulation	CreEL-PTX (N = 8)	nab-PTX (N = 7)	micellar-PTX (N = 6)	CreEL-PTX (N = 8)	nab-PTX (N = 7)	micellar-PTX (N = 6)
Tissue						
Parameter	K_p,tissue_			K_p,uu,tissue_		
Brain (Br)	0.16 ± 0.07	0.44 ± 0.19	0.38 ± 0.06	0.006 ± 0.002	0.010 ± 0.004	0.008 ± 0.001
Spinal cord (SC)	0.11 ± 0.02	0.39 ± 0.09	0.41 ± 0.17	0.004 ± 0.001	0.009 ± 0.002	0.009 ± 0.003
Skeletal muscle (SM)	5.36 ± 3.04	14.76 ± 5.23	17.54 ± 2.45	1.680 ± 0.830	2.980 ± 0.920	3.300 ± 0.640
Sciatic nerve (SN)	2.24 ± 1.00	5.47 ± 3.52	7.12 ± 1.36	0.890 ± 0.350	1.410 ± 0.790	1.710 ± 0.380
Dorsal root ganglia (DRG)	2.10 ± 1.10	8.61 ± 2.56	7.83 ± 1.40	0.270 ± 0.123	0.710 ± 0.185	0.600 ± 0.116

Standard deviations of K_p,uu,tissue_ values were calculated using the error propagation method (Loryan et al., 2017). N, number of animals.

**FIGURE 2:**
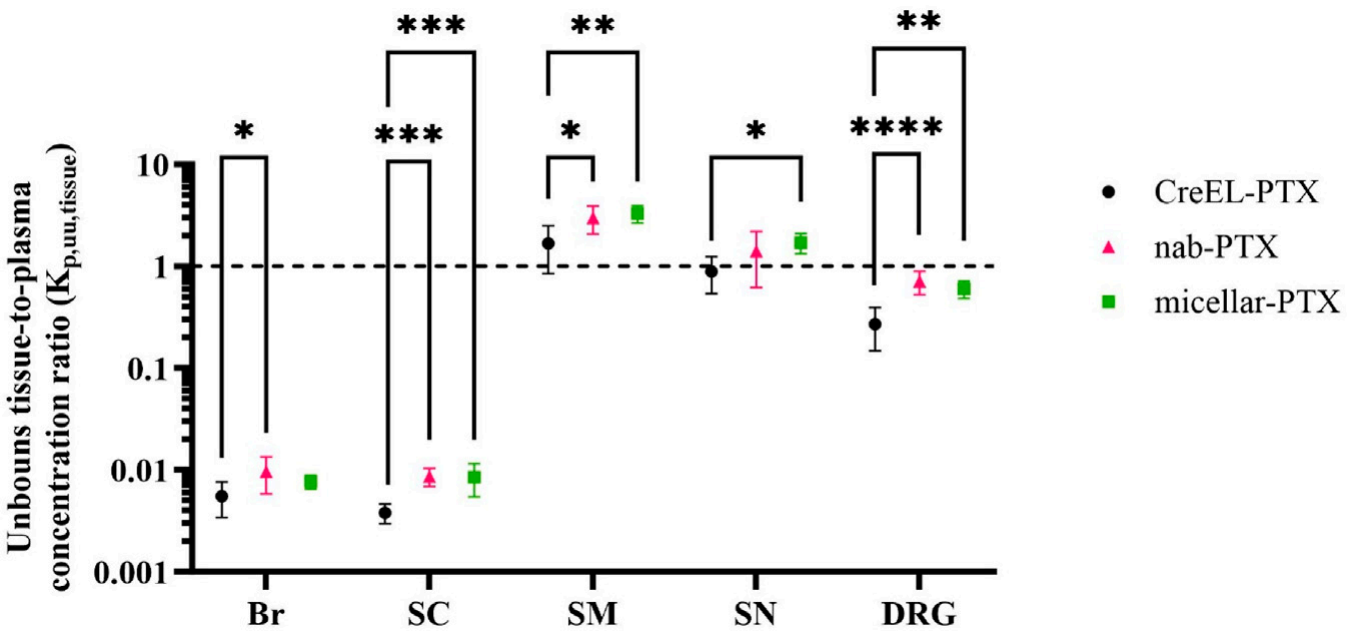
Unbound paclitaxel tissue distribution after administration of CreEL-PTX (N = 8, black), nab-PTX (N = 7, magenta) and micellar-PTX (N = 6, green) formulations. Semilogarithmic scatter plots of mean ± SD values of unbound paclitaxel tissue-to-plasma concentration ratios in the brain (Br), spinal cord (SC), skeletal muscle (SM), sciatic nerve (SN) and dorsal root ganglia (DRG). The dotted line indicates a K_p,uu,tissue_ of unity. The standard deviations were calculated using the error propagation method (Loryan et al., 2017). The comparisons were performed using one-way ANOVA followed by Tukey’s multiple comparisons within each tissue separately. Significance levels were <0.05 (*), <0.01 (**), <0.001 (***), <0.0001 (****).

The most notable difference between treatment arms was observed in the extent of unbound paclitaxel distribution across BDB, with K_p,uu,DRG_ of 0.27 after CreEL-PTX compared to 0.71 after nab-PTX (*p* < 0.0001), and 0.6 after micellar-PTX (*p* < 0.0014). Close to a 2-fold difference was observed in the extent of unbound paclitaxel distribution across BSCB between CreEL-PTX, with a K_p,uu,SC_ of 0.004 versus 0.009 in the nab-PTX (*p* = 0.0005) and micellar-PTX (*p* = 0.0009) arms (Figure 2 and Table 4).

### 3.4 Total paclitaxel distribution to CIPN-sites is higher after administration of nab- and micellar-PTX compared to CreEL-PTX

In addition to studying the net flux of paclitaxel across the barriers, it is also important to understand total paclitaxel exposure in the CIPN-sites, which is estimated using K_p,tissue_ when total plasma exposure is known. This helps to evaluate the accumulation of paclitaxel in the nervous system, which may lead to CIPN development. Therefore, the total tissue-to-plasma concentration ratios were also compared (Equation 7).

**FIGURE 3:**
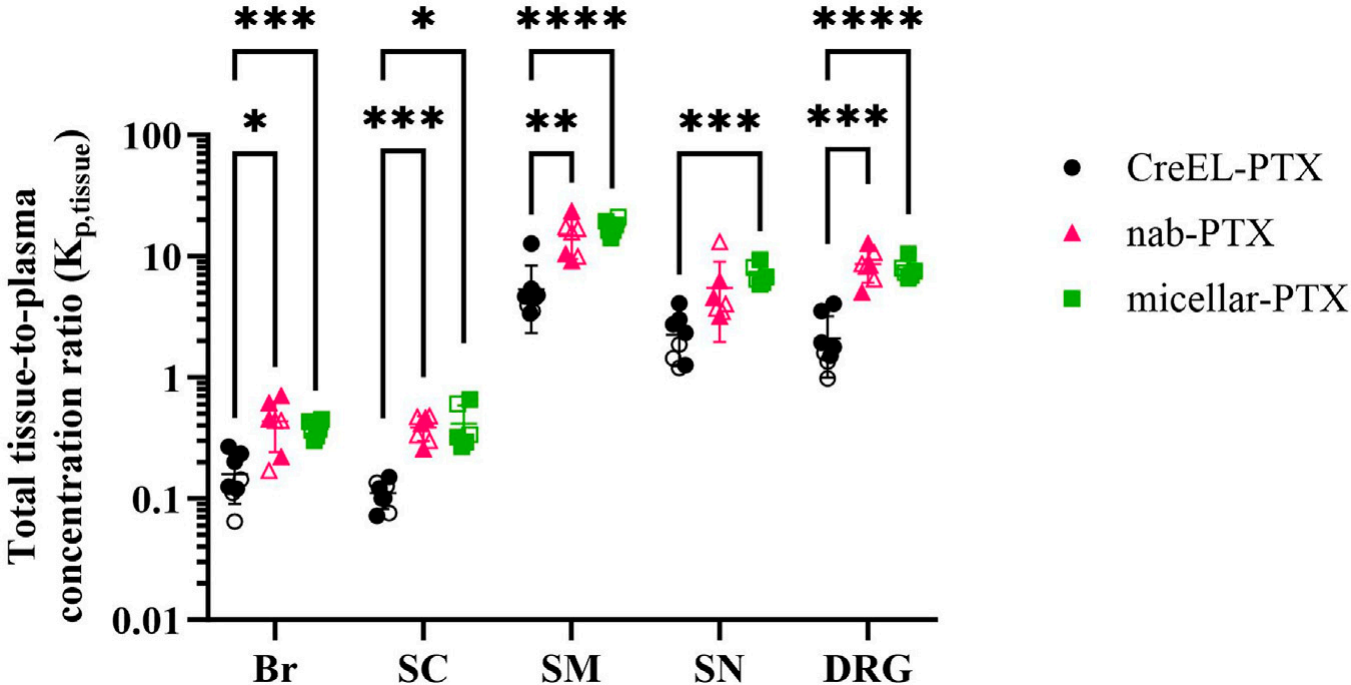
Total paclitaxel tissue distribution after administration of a 4-h intravenous infusion of 4 mg/kg CreEL-PTX (N = 5 males (M), 3 females (F), black), 4 mg/kg nab-PTX (N = 3M, 4F, magenta) and 1 mg/kg micellar-PTX (N = 4M, 2F, green) formulations. Semilogarithmic scatter dot plot of total paclitaxel tissue-to-plasma concentration ratios in male (filled symbols) and female (open symbols) rats in the brain (Br), spinal cord (SC), skeletal muscle (SM), sciatic nerve (SN) and dorsal root ganglia (DRG). Data presented as mean ± SD. K_p,tissue_ comparison between treatment arms was performed using a two-way ANOVA test followed by Tukey’s multiple comparisons. Significance levels are depicted as follows: <0.05 (*), <0.01 (**), <0.001 (***), <0.0001 (****).

No differences in K_p,tissue_ between male and female rats were identified in any of the treatment arms (Figure 3 filled versus open symbols). Therefore, animals of both sexes were included in the respective treatment arms for the comparison of K_p,tissue_ ratios between formulations. At least 2-fold higher K_p,SN_ and K_p,DRG_ values in the nab- and micellar-PTX arms compared to the CreEL-PTX arm showed an increased total paclitaxel exposure in the sciatic nerve and DRG compared to plasma in respective treatment arms (Table 4; Figure 3). The highest extent of total paclitaxel distribution was observed in the skeletal muscle, with mean K_p,SM_ of 5.36, 14.76 and 17.54 in the CreEL-, nab- and micellar-PTX arms, respectively. In line with the unbound ratios, the total paclitaxel exposure was at least 10-fold lower in the CNS tissues compared to the PNS tissues in all treatment arms (Table 4).

No difference in K_p,tissue_ was identified between the nab- and micellar-PTX arms in any of the tissues. However, significant differences were found between the CreEL-PTX arm in comparison to the Cremophor-free formulations. The most prominent differences in K_p,tissue_ values were observed in K_p,DRG_ of 2.1 and 7.8 (*p* < 0.0001), and K_p,SM_ of 5.4 and 17.5 (*p* < 0.0001), between CreEL-PTX and micellar-PTX arms, respectively. Similarly, a significant difference was observed in K_p,DRG_ when comparing CreEL-PTX with nab-PTX arms, where K_p,DRG_ were 2.1 and 8.6 (*p* = 0.0007), respectively (Figure 3; Table 4).

### 3.5 Dexamethasone does not change the extent of paclitaxel transport across barriers

**FIGURE 4:**
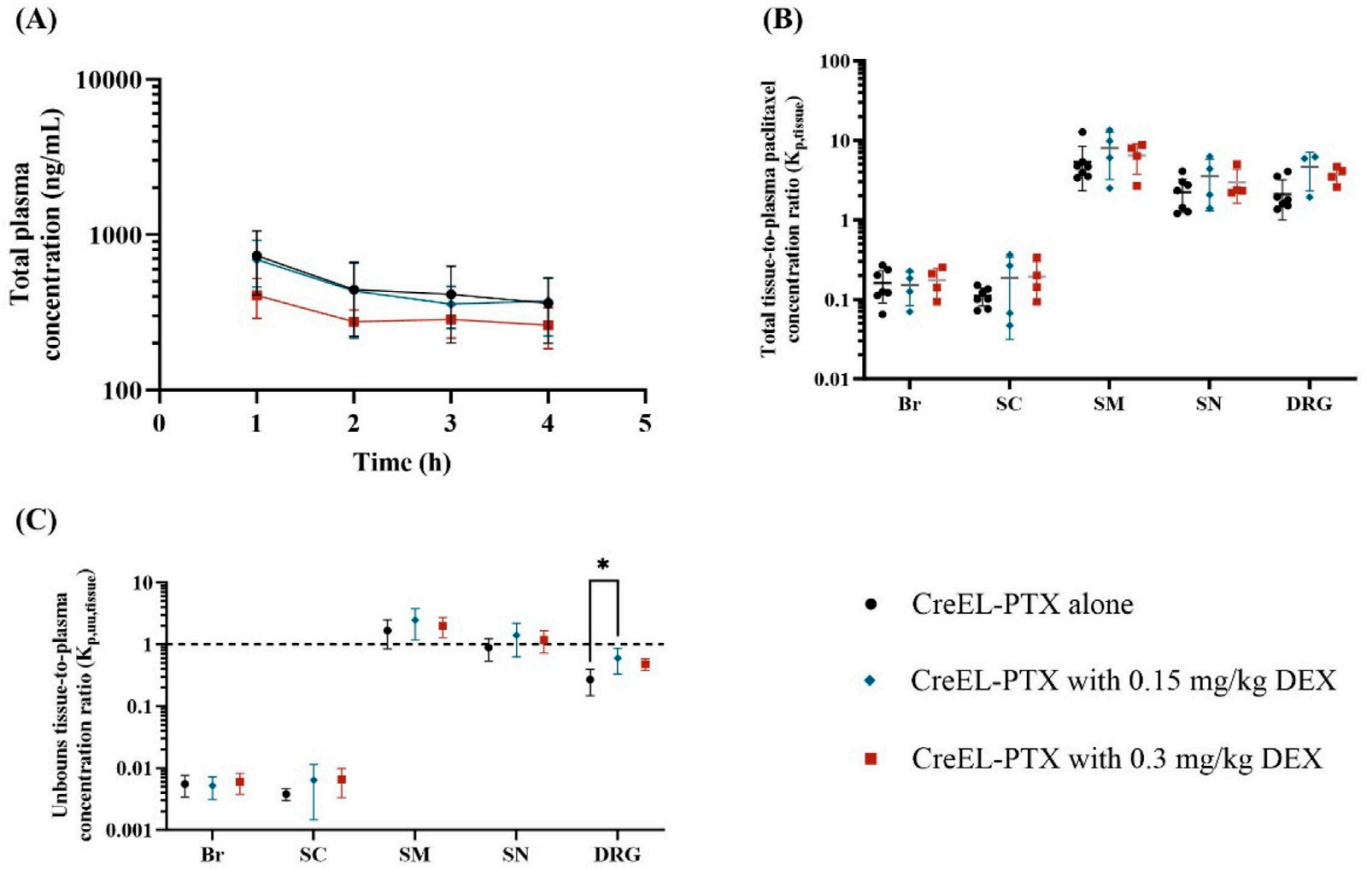
The impact of dexamethasone premedication on total plasma exposure and the extent of total and unbound paclitaxel distribution after a 4-h IV infusion of 4 mg/kg CreEL-PTX alone (n = 8, black), with 0.15 mg/kg DEX (n = 4, blue) and with 0.3 mg/kg DEX (n = 4, red) premedication. **(A)** semilogarithmic plot represents total paclitaxel plasma concentration (ng/mL) over time, **(B)** semilogarithmic scatter dot plot of total paclitaxel tissue-to-plasma concentration ratios in male rats, and **(C)** semilogarithmic scatter plot of mean ± SD values of unbound paclitaxel tissue-to-plasma concentration ratios in brain (Br), spinal cord (SC), skeletal muscle (SM), sciatic nerve (SN) and dorsal root ganglia (DRG). Data are presented as mean ± SD. K_p,tissue_ comparison between treatment arms was performed using a two-way ANOVA test followed by Tukey’s multiple comparisons. The dotted line in C indicates a K_p,uu,tissue_ of unity. The standard deviation was calculated using the error propagation method (Loryan et al., 2017). For K_p,uu,tissue_ comparison was performed using a one-way ANOVA test followed by Tukey’s multiple comparisons within each tissue separately. Significance levels are depicted as follows: <0.05 (*), <0.01 (**), <0.001 (***), <0.0001 (****). Abbreviation: DEX - dexamethasone.

No significant differences were found in total paclitaxel plasma concentrations between the CreEL-PTX treatment arms with or without dexamethasone pretreatment (Figure 4A), although there is a tendency towards lower concentrations for the highest dose of dexamethasone. At the end of the infusion, the plasma concentration of paclitaxel was 362 ± 162 ng/mL, 375 ± 152 ng/mL, and 262 ± 77 ng/mL for CreEL-PTX alone, with 0.15 mg/kg DEX and with 0.3 mg/kg DEX, respectively.

There were no differences in the extent of total paclitaxel distribution across treatment arms in the different tissues (Figure 4B). The only difference in K_p,uu,tissue_ values was observed in K_p,uu,DRG_ between the CreEL-PTX alone arm and pretreatment with 0.15 mg/kg DEX, with K_p,uu,DRG_ of 0.27 and 0.60 (*p* = 0.019), respectively (Figure 4C; Supplementary Table S4).

## 4 Discussion

We here characterize the impact of three clinically used paclitaxel formulations, CreEL-PTX, nab-PTX, and micellar-PTX on paclitaxel distribution to CIPN-sites, which may shed light on pharmacokinetic mechanisms of documented dissimilarities in CIPN prevalence in patients receiving different formulations. This was achieved by using the CMA-CIPN approach (Hu et al., 2024), comparing total (K_p,tissue_) and unbound (K_p,uu,tissue_) paclitaxel partition coefficients across the endothelial membranes to CIPN-sites after a 4-h intravenous infusion. We found that the nab- and micellar-PTX formulations on average showed up to three-fold higher total and unbound paclitaxel tissue-to-plasma ratios in comparison with CreEL-PTX. On the other hand, dexamethasone, commonly used for premedication before CreEL-PTX, had nearly no impact on the tissue distribution of paclitaxel after the CreEL-PTX treatment, but a significant difference in the distribution to the DRG between treatment arms was observed.

To understand the extent of paclitaxel distribution to CIPN-sites, K_p,uu,tissue_ was used to determine if active uptake (K_p,uu,tissue_ > 1) or efflux (K_p,uu,tissue_ < 1) processes across the barriers are predominant (Hammarlund-Udenaes et al., 2008). Across all paclitaxel formulations, we observed a higher extent of paclitaxel distribution to the sciatic nerve, DRG as well as skeletal muscles compared to the brain and spinal cord. These results can be explained by the known structural, biochemical, and functional differences between barriers, including paracellular transport (Hu et al., 2024) and expression of transporters. In *in vitro* studies, paclitaxel has been shown to be a substrate for both active uptake and efflux transporters. Paclitaxel transport is mediated by transporters such as solute carrier organic anion-transporting polypeptide B1 and B3 (OATP1B1, OATP1B3) (Leblanc et al., 2018; Svoboda et al., 2011), organic anion transporter 2 (OAT2) (Kobayashi et al., 2005), and P-glycoprotein (ABCB1, P-gp) (Fellner et al., 2002). The mean unbound partitioning, described by K_p,uu,br_ and K_p,uu,sc_ was in all treatment arms found to be lower than 0.01. This can be explained by the tightness of the BBB and BSCB compared to PNS barriers, together with a high expression of P-gp in the CNS barriers (Fellner et al., 2002). In the case of BNB and BDB, we estimated K_p,uu,SN_ and K_p,uu,DRG_ values to be around or even above unity suggesting an active uptake to be predominant at the barrier site, which may be a potential factor of CIPN development. Higher than unity K_p,uu,SN_ and K_p,uu,DRG_ values also were observed after 10 days of CreEL-PTX IV infusion (Hu et al., 2024). The importance of active uptake transporters in CIPN development was observed when a deficiency of Oatp1b2 in a mice model led to a decreased paclitaxel uptake into DRG and a lack of development of CIPN-related symptoms in mice (Leblanc et al., 2018).

Previously CreEL was shown to inhibit active uptake transporters (human OATP1B1, OATP1B3, and rat Oatp1b2) at clinically relevant concentrations *in vitro*, reducing paclitaxel transport into cells that were transfected with specific transporters (Nieuweboer et al., 2014). This suggests a possible mechanism through which formulations can impact drug transport across barriers *in vivo*. By comparing total and unbound paclitaxel tissue-to-plasma ratios at CIPN-sites between paclitaxel formulations, we were able to show that the nab- and micellar-PTX formulations on average exhibited up to three-fold higher total and unbound partitioning coefficients in comparison to CreEL-PTX (Table 4). These findings provide new evidence that the presence of CreEL in the paclitaxel formulation leads to lower paclitaxel accumulation and transport across the investigated barriers also *in vivo*, compared to Cremophor-free formulations.

In addition to the differences in the extent of paclitaxel distribution to CIPN-sites between formulations, we also observed differences in the fraction of unbound paclitaxel in plasma. We have documented a two-fold lower f_u,plasma_ in the CreEL-PTX arm in comparison to nab- and micellar-PTX (Table 3). Our results were in line with pharmacokinetic data reported in humans, with the lowest f_u,plasma_ of paclitaxel in the CreEL-PTX arm (Table 1). The effect of CreEL on the fraction of unbound paclitaxel in plasma has been explored using pharmacokinetic mathematical modeling showing paclitaxel binding being proportional to the CreEL concentration (Karlsson et al., 1999; Henningsson et al., 2001). In particular, it has been shown that higher CreEL concentrations in plasma were associated with a lower free fraction of paclitaxel (Henningsson et al., 2001). Therefore, the lack of CreEL in nab- and micellar-PTX formulations in plasma may explain their higher f_u,plasma_. This has also been seen in humans, with f_u,plasma_ up to 0.062 in both nab- and micellar-PTX-treated patients (Borgå et al., 2019a). In our study, differences in paclitaxel plasma protein binding between formulations have been also reflected in almost 10-fold higher total paclitaxel concentrations in plasma in the CreEL-PTX arm compared to the micellar-PTX arm (Figure 1A). In this study to discriminate the differences between paclitaxel formulations in active transport processes it was also important to study the unbound drug distribution across the barriers. It is worth to note, that in the case of differences in plasma binding between formulations, ideally similar unbound concentrations should be targeted to exclude other potential factors affecting pharmacokinetic processes.

In contrast to the differences in tissue distribution across formulations, we found that the selected dexamethasone doses had nearly no impact on the distribution of paclitaxel into CIPN-sites after CreEL-PTX treatment, except the dexamethasone premedication with the dose of 0.15 mg/kg leading to 2-fold higher K_p,uu,DRG_ compared to the CreEL-PTX alone. In addition, no significant changes were observed neither in total plasma concentrations or in plasma protein binding. Therefore, we hypothesize that premedication with dexamethasone itself will less likely increase the pharmacokinetic-related probability of CIPN development.

We determined paclitaxel distribution to CIPN-sites between different formulations using the CMA-CIPN approach (Hu et al., 2024). The approach among other benefits reduces the required number of animals, in comparison to the conventional approach of tissue partitioning determination using overall exposure in plasma and tissues measured by the total area under the concentration curves (Hammarlund-Udenaes et al., 2008). An AUC-based approach was used by Li et al. to determine paclitaxel exposure in the brain, skeletal muscle, and other tissues in female mice after 10 mg/kg bolus intravenous administration of different paclitaxel formulations, including CreEL-, nab- and micellar-PTX (Li et al., 2018). A similar tendency of more than 4-fold lower paclitaxel systemic exposure in plasma and at least 2-fold higher K_p,Br_ and K_p,SM_ values in CreEL-free formulations compared to the CreEL-PTX arm were observed. However, absolute values cannot be compared between Li et al. work and the results presented in this study, due to differences in infusion and sampling time. In another study, an AUC-based approach was used to determine the total extent of paclitaxel distribution to the sciatic nerve and DRG after bolus injection of 30 mg/kg of CreEL-PTX in female mice (Wozniak et al., 2016). Until the present study, only total K_p,tissue_ values were determined for nab-PTX and micellar-PTX. K_p,tissue_ values may be used to understand overall paclitaxel exposure in the tissues and its association with CIPN development, but not the net flux of undergoing transport mechanisms across the barriers, which can be an important factor of CIPN development. To fully understand the mechanisms of the CIPN development, the differences in the net flux through the barriers between formulations, shown by unbound K_p,uu,tissue_ values, are required, because the unbound partition coefficient reflects changes occurring on the level of the net flux across blood-to-tissue barriers.

Here we focused on identifying possible differences in the extent of total and unbound paclitaxel distribution to CIPN-sites after one cycle of paclitaxel infusion. Our observations highlight the importance of conducting CIPN-specific paclitaxel distribution studies with novel formulations, to characterize their safety profiles, which may differ due to the used excipient. These results can deepen the understanding of CIPN development and provide new insights on how to monitor and adjust paclitaxel treatment with different paclitaxel formulations, to lower the risk of CIPN.

## 5 Conclusion

The evaluation of paclitaxel distribution to CIPN-sites after administration of three clinically used paclitaxel formulations provides intricate tissue-specific distribution patterns that may influence CIPN development. Based on our data, the Cremophor-free paclitaxel formulations nab- and micellar-PTX, are on average associated with a higher distribution of paclitaxel to CIPN-sites than CreEL-PTX. By evaluating unbound drug disposition at conventional and non-conventional CIPN-sites, differences in active transport processes were determined between paclitaxel formulations. Together with similar unbound paclitaxel exposure in plasma and assumed species-independent extent of paclitaxel transport across the barriers, nab- and micellar-PTX formulations may lead to higher paclitaxel exposure at CIPN-sites in comparison to CreEL-PTX. Further pharmacokinetic study with repeated nab- and micellar-PTX dosing including CIPN evaluation is needed to verify the pharmacokinetics-driven mechanism predisposing CIPN development.

## Data Availability

The raw data supporting the conclusions of this article will be made available by the authors, without undue reservation.

## References

[B1] Anderson C. D. Wang J. Kumar G. N. McMillan J. M. Walle U. K. Walle T. (1995). Dexamethasone induction of taxol metabolism in the rat. Drug Metab. Dispos. 23 (11), 1286–1290. 8591732

[B2] Balayssac D. Busserolles J. Broto C. Dalbos C. Prival L. Lamoine S. (2023). Neurofilament light chain in plasma as a sensitive diagnostic biomarker of peripheral neurotoxicity: *in vivo* mouse studies with oxaliplatin and paclitaxel - NeuroDeRisk project. Biomed. Pharmacother. = Biomedecine Pharmacother. 167, 115535. 10.1016/j.biopha.2023.115535 37738793

[B3] Barroso A. Estevinho F. Hespanhol V. Teixeira E. Ramalho-Carvalho J. Araújo A. (2024). Management of infusion-related reactions in cancer therapy: strategies and challenges. ESMO Open 9 (3), 102922. 10.1016/j.esmoop.2024.102922 38452439 PMC10937241

[B4] Bashir Q. Acosta M. (2020). Comparative safety, bioavailability, and pharmacokinetics of oral dexamethasone, 4-mg and 20-mg tablets, in healthy volunteers under fasting and fed conditions: a randomized open-label, 3-way crossover study. Clin. Lymphoma, Myeloma Leuk. 20 (11), 768–773. 10.1016/j.clml.2020.06.022 32900662

[B5] Borgå O. Henriksson R. Bjermo H. Lilienberg E. Heldring N. Loman N. (2019b). Maximum tolerated dose and pharmacokinetics of paclitaxel micellar in patients with recurrent malignant solid tumours: a dose-escalation study. Adv. Ther. 36 (5), 1150–1163. 10.1007/s12325-019-00909-6 30879251 PMC6824363

[B6] Borgå O. Lilienberg E. Bjermo H. Hansson F. Heldring N. Dediu R. (2019a). Pharmacokinetics of total and unbound paclitaxel after administration of paclitaxel micellar or nab-paclitaxel: an open, randomized, cross-over, explorative study in breast cancer patients. Adv. Ther. 36 (10), 2825–2837. 10.1007/s12325-019-01058-6 31432461 PMC6822820

[B7] Delaloge S. Pérol D. Courtinard C. Brain E. Asselain B. Bachelot T. (2016). Paclitaxel plus bevacizumab or paclitaxel as first-line treatment for HER2-negative metastatic breast cancer in a multicenter national observational study. Ann. Oncol. 27 (9), 1725–1732. 10.1093/annonc/mdw260 27436849

[B8] Di L. Umland J. P. Trapa P. E. Maurer T. S. (2012). Impact of recovery on fraction unbound using equilibrium dialysis. J. Pharm. Sci. 101 (3), 1327–1335. 10.1002/jps.23013 22161810

[B9] Dubinsky S. Patel D. Wang X. Srikanthan A. Ng T. L. Tsang C. (2022). Pre-medication protocols for the prevention of paclitaxel-induced infusion related reactions: a systematic review and meta-analysis. Support Care Cancer 30 (7), 5627–5644. 10.1007/s00520-022-06891-0 35150312

[B10] EMA (2022). ICH guidelines.

[B11] EMA (2024). Public statement on apealea: withdrawal of the marketing authorisation in the European union. Amsterdam, Netherlands: European Medicines Agency.

[B12] FDA (2022). M10 bioanalytical method validation and study sample analysis. Silver Spring, MD, USA: Food and Drug Administration.

[B13] Fellner S. Bauer B. Miller D. S. Schaffrik M. Fankhänel M. Spruss T. (2002). Transport of paclitaxel (Taxol) across the blood-brain barrier *in vitro* and *in vivo* . J. Clin. Invest. 110 (9), 1309–1318. 10.1172/JCI15451 12417570 PMC151606

[B14] Gelderblom H. Verweij J. Nooter K. Sparreboom A. (2001). Cremophor EL: the drawbacks and advantages of vehicle selection for drug formulation. Eur. J. Cancer (Oxford, Engl. 1990) 37 (13), 1590–1598. 10.1016/s0959-8049(01)00171-x 11527683

[B15] Hammarlund-Udenaes M. Fridén M. Syvänen S. Gupta A. (2008). On the rate and extent of drug delivery to the brain. Pharm. Res. 25 (8), 1737–1750. 10.1007/s11095-007-9502-2 18058202 PMC2469271

[B16] Henningsson A. Karlsson M. O. Viganò L. Gianni L. Verweij J. Sparreboom A. (2001). Mechanism-based pharmacokinetic model for paclitaxel. J. Clin. Oncol. 19 (20), 4065–4073. 10.1200/JCO.2001.19.20.4065 11600609

[B17] Hu Y. Girdenyté M. Roest L. Liukkonen I. Siskou M. Bällgren F. (2024). Analysis of the contributing role of drug transport across biological barriers in the development and treatment of chemotherapy-induced peripheral neuropathy. Fluids Barriers CNS 21 (1), 13. 10.1186/s12987-024-00519-7 38331886 PMC10854123

[B18] Hwang B. Y. Kim E. S. Kim C. H. Kwon J. Y. Kim H. K. (2012). Gender differences in paclitaxel-induced neuropathic pain behavior and analgesic response in rats. Korean J. Anesthesiol. 62 (1), 66–72. 10.4097/kjae.2012.62.1.66 22323957 PMC3272532

[B19] Kalvass J. C. Maurer T. S. (2002). Influence of nonspecific brain and plasma binding on CNS exposure: implications for rational drug discovery. Biopharm. Drug Dispos. 23 (8), 327–338. 10.1002/bdd.325 12415573

[B20] Karam A. Ledermann J. A. Kim J. W. Sehouli J. Lu K. Gourley C. (2017). Fifth ovarian cancer consensus conference of the gynecologic cancer InterGroup: first-line interventions. Ann. Oncol. 28 (4), 711–717. 10.1093/annonc/mdx011 28327917

[B21] Karlsson M. O. Molnar V. Freijs A. Nygren P. Bergh J. Larsson R. (1999). Pharmacokinetic models for the saturable distribution of paclitaxel. Drug Metab. Dispos. 27 (10), 1220–1223. 10497151

[B22] Kobayashi Y. Ohshiro N. Sakai R. Ohbayashi M. Kohyama N. Yamamoto T. (2005). Transport mechanism and substrate specificity of human organic anion transporter 2 (hOat2 [SLC22A7]). J. Pharm. Pharmacol. 57 (5), 573–578. 10.1211/0022357055966 15901346

[B23] Leblanc A. F. Sprowl J. A. Alberti P. Chiorazzi A. Arnold W. D. Gibson A. A. (2018). OATP1B2 deficiency protects against paclitaxel-induced neurotoxicity. J. Clin. Invest. 128 (2), 816–825. 10.1172/JCI96160 29337310 PMC5785270

[B24] Li F. Zhang H. He M. Liao J. Chen N. Li Y. (2018). Different nanoformulations alter the tissue distribution of paclitaxel, which aligns with reported distinct efficacy and safety profiles. Mol. Pharm. 15 (10), 4505–4516. 10.1021/acs.molpharmaceut.8b00527 30180593 PMC8851508

[B25] Liu M. Liu S. Yang L. Wang S. (2021). Comparison between nab-paclitaxel and solvent-based taxanes as neoadjuvant therapy in breast cancer: a systematic review and meta-analysis. BMC Cancer 21 (1), 118. 10.1186/s12885-021-07831-7 33541289 PMC7863369

[B26] Loryan I. Hoppe E. Hansen K. Held F. Kless A. Linz K. (2017). Quantitative assessment of drug delivery to tissues and association with phospholipidosis: a case study with two structurally related diamines in development. Mol. Pharm. 14 (12), 4362–4373. 10.1021/acs.molpharmaceut.7b00480 29099189

[B27] Loryan I. Sinha V. Mackie C. Van Peer A. Drinkenburg W. Vermeulen A. (2014). Mechanistic understanding of brain drug disposition to optimize the selection of potential neurotherapeutics in drug discovery. Pharm. Res. 31 (8), 2203–2219. 10.1007/s11095-014-1319-1 24623476

[B28] McMahon D. Oakden W. Hynynen K. (2020). Investigating the effects of dexamethasone on blood-brain barrier permeability and inflammatory response following focused ultrasound and microbubble exposure. Theranostics 10 (4), 1604–1618. 10.7150/thno.40908 32042325 PMC6993222

[B29] Mielke S. Sparreboom A. Steinberg S. M. Gelderblom H. Unger C. Behringer D. (2005). Association of Paclitaxel pharmacokinetics with the development of peripheral neuropathy in patients with advanced cancer. Clin. Cancer Res. 11 (13), 4843–4850. 10.1158/1078-0432.CCR-05-0298 16000582

[B30] Naji-Esfahani H. Vaseghi G. Safaeian L. Pilehvarian A.-A. Abed A. Rafieian-Kopaei M. (2016). Gender differences in a mouse model of chemotherapy-induced neuropathic pain. Lab. Anim. 50 (1), 15–20. 10.1177/0023677215575863 25732574

[B31] Nakamura I. Ichimura E. Goda R. Hayashi H. Mashiba H. Nagai D. (2017). An *in vivo* mechanism for the reduced peripheral neurotoxicity of NK105: a paclitaxel-incorporating polymeric micellar nanoparticle formulation. Int. J. Nanomedicine 12, 1293–1304. 10.2147/IJN.S114356 28243090 PMC5317268

[B32] Nieuweboer A. J. Hu S. Gui C. Hagenbuch B. Ghobadi Moghaddam-Helmantel I. M. Gibson A. A. (2014). Influence of drug formulation on OATP1B-mediated transport of paclitaxel. Cancer Res. 74 (11), 3137–3145. 10.1158/0008-5472.CAN-13-3634 24755470 PMC4161133

[B33] Omran M. Belcher E. K. Mohile N. A. Kesler S. R. Janelsins M. C. Hohmann A. G. (2021). Review of the role of the brain in chemotherapy-induced peripheral neuropathy. Front. Mol. Biosci. 8, 693133. 10.3389/fmolb.2021.693133 34179101 PMC8226121

[B34] Paksoy N. Khanmammadov N. Doğan İ. Ferhatoğlu F. Ahmed M. A. Karaman S. (2023). Weekly paclitaxel treatment in the first-line therapy of classic Kaposi sarcoma: a real-life study. Medicine 102 (5), e32866. 10.1097/MD.0000000000032866 36749246 PMC9901949

[B35] Pang D. Kocherginsky M. Krausz T. Kim S.-Y. Conzen S. D. (2006). Dexamethasone decreases xenograft response to Paclitaxel through inhibition of tumor cell apoptosis. Cancer Biol. Ther. 5 (8), 933–940. 10.4161/cbt.5.8.2875 16775428

[B36] Percie du Sert N. Hurst V. Ahluwalia A. Alam S. Avey M. T. Baker M. (2020). The ARRIVE guidelines 2.0: updated guidelines for reporting animal research. PLoS Biol. 18 (7), e3000410. 10.1371/journal.pbio.3000410 32663219 PMC7360023

[B37] Peters C. M. Jimenez-Andrade J. M. Kuskowski M. A. Ghilardi J. R. Mantyh P. W. (2007). An evolving cellular pathology occurs in dorsal root ganglia, peripheral nerve and spinal cord following intravenous administration of paclitaxel in the rat. Brain Res. 1168, 46–59. 10.1016/j.brainres.2007.06.066 17698044 PMC2042964

[B38] Samtani M. N. Jusko W. J. (2005). Comparison of dexamethasone pharmacokinetics in female rats after intravenous and intramuscular administration. Biopharm. Drug Dispos. 26 (3), 85–91. 10.1002/bdd.435 15654687 PMC4178533

[B39] Seretny M. Currie G. L. Sena E. S. Ramnarine S. Grant R. MacLeod M. R. (2014). Incidence, prevalence, and predictors of chemotherapy-induced peripheral neuropathy: a systematic review and meta-analysis. Pain 155 (12), 2461–2470. 10.1016/j.pain.2014.09.020 25261162

[B40] Sparreboom A. Scripture C. D. Trieu V. Williams P. J. De T. Yang A. (2005). Comparative preclinical and clinical pharmacokinetics of a cremophor-free, nanoparticle albumin-bound paclitaxel (ABI-007) and paclitaxel formulated in cremophor (taxol). Clin. Cancer Res. 11 (11), 4136–4143. 10.1158/1078-0432.CCR-04-2291 15930349

[B41] Summerfield S. G. Stevens A. J. Cutler L. del Carmen Osuna M. Hammond B. Tang S. P. (2006). Improving the *in vitro* prediction of *in vivo* central nervous system penetration: integrating permeability, P-glycoprotein efflux, and free fractions in blood and brain. J. Pharmacol. Exp. Ther. 316 (3), 1282–1290. 10.1124/jpet.105.092916 16330496

[B42] Summerfield S. G. Yates J. W. T. Fairman D. A. (2022). Free drug theory - No longer just a hypothesis? Pharm. Res. 39 (2), 213–222. 10.1007/s11095-022-03172-7 35112229

[B43] Svoboda M. Wlcek K. Taferner B. Hering S. Stieger B. Tong D. (2011). Expression of organic anion-transporting polypeptides 1B1 and 1B3 in ovarian cancer cells: relevance for paclitaxel transport. Biomed. Pharmacother. 65 (6), 417–426. 10.1016/j.biopha.2011.04.031 21719246

[B44] Tan H. Hu J. Liu S. (2019). Efficacy and safety of nanoparticle albumin-bound paclitaxel in non-small cell lung cancer: a systematic review and meta-analysis. Artif. Cells, Nanomedicine, Biotechnol. 47 (1), 268–277. 10.1080/21691401.2018.1552595 30600739

[B45] Vergote I. Bergfeldt K. Franquet A. Lisyanskaya A. S. Bjermo H. Heldring N. (2020). A randomized phase III trial in patients with recurrent platinum sensitive ovarian cancer comparing efficacy and safety of paclitaxel micellar and Cremophor EL-paclitaxel. Gynecol. Oncol. 156 (2), 293–300. 10.1016/j.ygyno.2019.11.034 31826802

[B46] Was H. Borkowska A. Bagues A. Tu L. Liu J. Y. H. Lu Z. (2022). Mechanisms of chemotherapy-induced neurotoxicity. Front. Pharmacol. 13, 750507. 10.3389/fphar.2022.750507 35418856 PMC8996259

[B47] Wozniak K. M. Vornov J. J. Wu Y. Nomoto K. Littlefield B. A. DesJardins C. (2016). Sustained accumulation of microtubule-binding chemotherapy drugs in the peripheral nervous system: correlations with time course and neurotoxic severity. Cancer Res. 76 (11), 3332–3339. 10.1158/0008-5472.CAN-15-2525 27197173 PMC4891279

