# Supplementary material for: Formulation-dependent differences in paclitaxel distribution to anatomical sites relevant to chemotherapy-induced peripheral neuropathy
Source: Front Pharmacol. 2024 Nov 6;15:1486686. doi: 10.3389/fphar.2024.1486686 (PMC11576287; doi:10.3389/fphar.2024.1486686)
Supplement: Supplementary file 1 [file Table1.pdf]

Table S1. Statistical test details of comparison of $K_{p,tissue}$ by paclitaxel formulation

	Mean 1	Mean 2	Differences between the means, Mean Diff.	Standard error of the Mean Diff.	N1	N2	Ratio reported by the Tukey's test, q	Degrees of freedom, DF	95% confidence interval of the Mean Diff.	Adjusted p value
Two-way ANOVA										<0.0001
$K_{p,Br}$										
CreEL-PTX vs. nab-PTX	0.159	0.436	-0.276	0.077	8	7	5.097	7.367	-0.499 to -0.053	0.0192
CreEL-PTX vs. micellar-PTX	0.159	0.376	-0.217	0.034	8	6	8.993	11.800	-0.308 to -0.126	0.0001
nab-PTX vs. micellar-PTX	0.436	0.376	0.060	0.076	7	6	1.106	7.238	-0.163 to 0.283	0.7247
$K_{p,SC}$										
CreEL-PTX vs. nab-PTX	0.111	0.389	-0.278	0.036	8	7	11.00	7.003	-0.383 to -0.173	0.0003
CreEL-PTX vs. micellar-PTX	0.111	0.414	-0.304	0.070	8	6	6.112	5.204	-0.529 to -0.078	0.0159
nab-PTX vs. micellar-PTX	0.389	0.414	-0.026	0.078	7	6	0.467	7.368	-0.251 to 0.200	0.9422
$K_{p,SM}$										
CreEL-PTX vs. nab-PTX	5.364	14.760	-9.397	2.250	8	7	5.905	9.364	-15.640 to -3.158	0.0056
CreEL-PTX vs. micellar-PTX	5.364	17.530	-12.170	1.468	8	6	11.720	11.890	-16.090 to -8.248	<0.0001
nab-PTX vs. micellar-PTX	14.76	17.530	-2.774	2.217	7	6	1.770	8.778	-8.991 to 3.444	0.4558
$K_{p,SN}$										
CreEL-PTX vs. nab-PTX	2.242	5.472	-3.230	1.376	8	7	3.320	6.855	-7.303 to 0.8429	0.1149
CreEL-PTX vs. micellar-PTX	2.242	7.116	-4.874	0.660	8	6	10.440	8.846	-6.722 to -3.025	0.0001
nab-PTX vs. micellar-PTX	5.472	7.116	-1.644	1.441	7	6	1.613	7.992	-5.762 to 2.475	0.5180
$K_{p,DRG}$										
CreEL-PTX vs. nab-PTX	2.097	8.606	-6.509	1.043	8	7	8.828	7.919	-9.495 to -3.523	0.0007

CreEL-PTX vs. micellar-PTX	2.097	7.827	-5.730	0.690	8	6	11.750	9.305	-7.645 to -3.816	<0.0001
nab-PTX vs. micellar-PTX	8.606	7.827	0.779	1.123	7	6	0.981	9.510	-2.325 to 3.883	0.7726

$K_{p,tissue}$ estimated using $C_{tot,tissue}$ and $C_{tot,plasma,ss}$ (Eq. 6). Data are presented in Fig. 3 and Table 4. The tests used were a matched two-way ANOVA followed by a Tukey's multiple comparison tests. Abbreviations: ANOVA – analysis of variance, br – brain, SC – spinal cord, SM – skeletal muscle, SN – sciatic nerve, DRG – dorsal root ganglia.

Table S2. Statistical test details of comparison of $K_{p,uu,tissue}$ by paclitaxel formulation

	Mean 1	Mean 2	Differences between the means, Mean Diff.	Standard error of the Mean Diff.	N1	N2	Ratio reported by the Tukey's test, q	Degrees of freedom, DF	95% confidence interval of the Mean Diff.	Adjusted p value
$K_{p,uu,Br}$										
One-way ANOVA										0.0251
CreEL-PTX vs. nab-PTX	0.0055	0.0096	-0.004	0.0014	8	7	4.256	18	-0.008 to -0.001	0.0196
CreEL-PTX vs. micellar-PTX	0.0055	0.0077	-0.002	0.0014	8	6	2.189	18	-0.006 to 0.001	0.2933
nab-PTX vs. micellar-PTX	0.0096	0.0077	0.002	0.0015	7	6	1.835	18	-0.002 to 0.006	0.4146
$K_{p,uu,SC}$										
One-way ANOVA										0.0002
CreEL-PTX vs. nab-PTX	0.0038	0.0086	-0.005	0.0010	8	7	6.660	18	-0.007 to -0.002	0.0005
CreEL-PTX vs. micellar-PTX	0.0038	0.0085	-0.005	0.0011	8	6	6.250	18	-0.007 to -0.002	0.0009
nab-PTX vs. micellar-PTX	0.0086	0.0085	0.0001	0.0011	7	6	0.129	18	-0.003 to 0.003	0.9954
$K_{p,uu,SM}$										
One-way ANOVA										0.0032
CreEL-PTX vs. nab-PTX	1.680	2.980	-1.300	0.4217	8	7	4.360	18	-2.376 to -0.2238	0.0168
CreEL-PTX vs. micellar-PTX	1.680	3.300	-1.620	0.4400	8	6	5.207	18	-2.743 to -0.4970	0.0046
nab-PTX vs. micellar-PTX	2.980	3.300	-0.320	0.4533	7	6	0.9984	18	-1.477 to 0.8369	0.7631
$K_{p,uu,SN}$										
One-way ANOVA										0.0336
CreEL-PTX vs. nab-PTX	0.890	1.410	-0.5200	0.2815	8	7	2.613	18	-1.238 to 0.198	0.1829
CreEL-PTX vs. micellar-PTX	0.890	1.710	-0.8200	0.2937	8	6	3.948	18	-1.570 to -0.070	0.0308
nab-PTX vs. micellar-PTX	1.410	1.710	-0.3000	0.3026	7	6	1.402	18	-1.072 to 0.472	0.5914
$K_{p,uu,DRG}$										
One-way ANOVA										<0.0001

CreEL-PTX vs. nab-PTX	0.270	0.710	-0.4400	0.0751	8	7	8.291	18	-0.632 to -0.249	<0.0001
CreEL-PTX vs. micellar-PTX	0.270	0.600	-0.3300	0.0783	8	6	5.959	18	-0.5230 to -0.130	0.0014
nab-PTX vs. micellar-PTX	0.710	0.600	0.1100	0.0807	7	6	1.928	18	-0.096 to 0.316	0.3802

$K_{p,uu,tissue}$ estimated using Combinatory Mapping Approach for CIPN (Eq. 7). Data are presented in Fig. 2 and Table 4. The tests used were a matched by the tissue one-way ANOVA followed by a Tukey's multiple comparison tests. Abbreviations: ANOVA – analysis of variance, br – brain, SC – spinal cord, SM – skeletal muscle, SN – sciatic nerve, DRG – dorsal root ganglia.

Table S3. Statistical test details of comparison of $K_{p,tissue}$ by CreEL-PTX premedication arm.

	Mean 1	Mean 2	Differences between the means, Mean Diff.	Standard error of the Mean Diff.	N1	N2	Ratio reported by the Tukey's test, q	Degrees of freedom, DF	95% confidence interval of the Mean Diff.	Adjusted p value
Two-way ANOVA										0.5713
$K_{p,Br}$										
CreEL-PTX alone vs. CreEL-PTX with 0.15 mg/kg DEX	0.159	0.152	0.008	0.0421	8	4	0.252	6	-0.121 to 0.136	0.9827
CreEL-PTX alone vs. CreEL-PTX with 0.3 mg/kg DEX	0.159	0.174	-0.015	0.0432	8	4	0.491	6	-0.148 to 0.118	0.9365
CreEL-PTX with 0.15 mg/kg DEX vs. CreEL-PTX with 0.3 mg/kg DEX	0.152	0.174	-0.023	0.0494	4	4	0.645	6	-0.174 to 0.129	0.8937
$K_{p,SC}$										
CreEL-PTX alone vs. CreEL-PTX with 0.15 mg/kg DEX	0.111	0.186	-0.076	0.0780	8	4	1.369	3	-0.395 to 0.244	0.6407
CreEL-PTX alone vs. CreEL-PTX with 0.3 mg/kg DEX	0.111	0.194	-0.083	0.0538	8	4	2.181	3	-0.298 to 0.132	0.3846
CreEL-PTX with 0.15 mg/kg DEX vs. CreEL-PTX with 0.3 mg/kg DEX	0.186	0.194	-0.008	0.0937	4	4	0.113	5	-0.306 to 0.291	0.9965
$K_{p,SM}$										
CreEL-PTX alone vs. CreEL-PTX with 0.15 mg/kg DEX	5.36	7.97	-2.601	2.606	8	4	1.412	4	-11.610 to 6.412	0.6139

CreEL-PTX alone vs. CreEL-PTX with 0.3 mg/kg DEX	5.36	6.43	-1.066	1.7200	8	4	0.876	7	-6.158 to 4.027	0.8146
CreEL-PTX with 0.15 mg/kg DEX vs. CreEL-PTX with 0.3 mg/kg DEX	7.97	6.43	1.536	2.7280	4	4	0.796	5	-7.514 to 10.590	0.8450
$K_{p,SN}$										
CreEL-PTX alone vs. CreEL-PTX with 0.15 mg/kg DEX	2.24	3.53	-1.290	1.1690	8	4	1.561	4	-5.662 to 3.082	0.5654
CreEL-PTX alone vs. CreEL-PTX with 0.3 mg/kg DEX	2.24	2.978	-0.736	0.7636	8	4	1.362	5	-3.273 to 1.802	0.6300
CreEL-PTX with 0.15 mg/kg DEX vs. CreEL-PTX with 0.3 mg/kg DEX	3.53	2.978	0.555	1.3030	4	4	0.602	5	-3.700 to 4.810	0.9067
$K_{p,DRG}$										
CreEL-PTX alone vs. CreEL-PTX with 0.15 mg/kg DEX	2.097	4.682	-2.585	1.4300	8	3	2.557	2	-9.842 to 4.671	0.3377
CreEL-PTX alone vs. CreEL-PTX with 0.3 mg/kg DEX	2.097	3.728	-1.632	0.5908	8	4	3.906	7	-3.345 to 0.082	0.0608
CreEL-PTX with 0.15 mg/kg DEX vs. CreEL-PTX with 0.3 mg/kg DEX	4.682	3.728	0.954	1.4460	3	4	0.933	2	-6.137 to 8.045	0.8037

$K_{p,tissue}$ estimated using $C_{tot,tissue}$ and $C_{tot,plasma,ss}$ (Eq. 6). Data are presented in Fig. 4B. The tests used were a matched two-way ANOVA followed by a Tukey's multiple comparison tests. Abbreviations: ANOVA – analysis of variance, br – brain, SC – spinal cord, SM – skeletal muscle, SN – sciatic nerve, DRG – dorsal root ganglia.

Table S4. Statistical test details of comparison of $K_{p,uu,tissue}$ by CreEL-PTX premedication arm.

	Mean 1	Mean 2	Differences between the means, Mean Diff.	Standard error of the Mean Diff.	N1	N2	Ratio reported by the Tukey's test, q	Degrees of freedom, DF	95% confidence interval of the Mean Diff.	Adjusted p value
$K_{p,uu,Br}$										
One-way ANOVA										0.8652
CreEL-PTX vs. CreEL-PTX with 0.15 mg/kg DEX	0.0055	0.0052	0.0003	0.0013	8	4	0.3263	13	-0.003 to 0.004	0.9712
CreEL-PTX vs. CreEL-PTX with 0.3 mg/kg DEX	0.0055	0.0060	-0.0005	0.0013	8	4	0.5438	13	-0.004 to 0.003	0.9222
CreEL-PTX with 0.15 mg/kg DEX vs. CreEL-PTX with 0.3 mg/kg DEX	0.0052	0.0060	-0.0008	0.0015	4	4	0.7535	13	-0.005 to 0.003	0.8568
$K_{p,uu,SC}$										
One-way ANOVA										0.2169
CreEL-PTX vs. CreEL-PTX with 0.15 mg/kg DEX	0.0038	0.0064	-0.0026	0.0018	8	4	2.061	13	-0.007 to 0.002	0.3421
CreEL-PTX vs. CreEL-PTX with 0.3 mg/kg DEX	0.0038	0.0066	-0.0028	0.0018	8	4	2.220	13	-0.008 to 0.002	0.2928
CreEL-PTX with 0.15 mg/kg DEX vs. CreEL-PTX with 0.3 mg/kg DEX	0.0064	0.0066	-0.0002	0.0021	4	4	0.137	13	-0.006 to 0.005	0.9948
$K_{p,uu,SM}$										
One-way ANOVA										0.3983
CreEL-PTX vs. CreEL-PTX with 0.15 mg/kg DEX	1.680	2.490	-0.810	0.5777	8	4	1.983	13	-2.335 to 0.715	0.3683
CreEL-PTX vs. CreEL-PTX with 0.3 mg/kg DEX	1.680	2.010	-0.330	0.5777	8	4	0.808	13	-1.855 to 1.195	0.8375

CreEL-PTX with 0.15 mg/kg DEX vs. CreEL-PTX with 0.3 mg/kg DEX	2.490	2.010	0.480	0.6671	4	4	1.018	13	-1.281 to 2.241	0.7565
$K_{p,uu,SN}$										
One-way ANOVA										0.2605
CreEL-PTX vs. CreEL-PTX with 0.15 mg/kg DEX	0.890	1.410	-0.520	0.3106	8	4	2.367	13	-1.340 to 0.300	0.2517
CreEL-PTX vs. CreEL-PTX with 0.3 mg/kg DEX	0.890	1.190	-0.300	0.3106	8	4	1.366	13	-1.120 to 0.520	0.6103
CreEL-PTX with 0.15 mg/kg DEX vs. CreEL-PTX with 0.3 mg/kg DEX	1.410	1.190	0.220	0.3587	4	4	0.867	13	-0.727 to 1.167	0.8155
$K_{p,uu,DRG}$										
One-way ANOVA										0.0157
CreEL-PTX vs. CreEL-PTX with 0.15 mg/kg DEX	0.270	0.600	-0.330	0.1035	8	3	4.509	12	-0.606 to -0.054	0.0198
CreEL-PTX vs. CreEL-PTX with 0.3 mg/kg DEX	0.270	0.480	-0.210	0.0936	8	4	3.172	12	-0.460 to 0.040	0.1038
CreEL-PTX with 0.15 mg/kg DEX vs. CreEL-PTX with 0.3 mg/kg DEX	0.600	0.480	0.120	0.1168	3	4	1.453	12	-0.192 to 0.432	0.5744

$K_{p,uu,tissue}$ estimated using Combinatory Mapping Approach for CIPN (Eq. 7). Data are presented in Fig. 4C. The tests used were a matched by the tissue one-way ANOVA followed by a Tukey’s multiple comparison tests. Abbreviations: ANOVA – analysis of variance, br – brain, SC – spinal cord, SM – skeletal muscle, SN – sciatic nerve, DRG – dorsal root ganglia.

Table S5. Summary of the steady-state total plasma concentrations ($C_{tot,ss,plasma}$), the steady-state unbound plasma concentrations ($C_{u,ss,plasma}$) and tissue concentrations ($C_{tot,tissue}$) after administration of 4 mg/kg CreEL-PTX (N=8), 4 mg/kg nab-PTX (N=7) and 1 mg/kg micellar-PTX (N=6) formulations. Data presented as mean ± SD.

Treatment arm	$C_{tot,ss,plasma}$ (ng/mL)	$C_{u,ss,plasma}$ (ng/mL)	$C_{tot,br}$ (ng/mL)	$C_{tot,SC}$ (ng/mL)	$C_{tot,SM}$ (ng/mL)	$C_{tot,SN}$ (ng/mL)	$C_{tot,DRG}$ (ng/mL)
CreEL-PTX	369.2 ± 124	14.4 ± 4.8	56.0 ± 28.5	42.1 ± 21.4	1889.4 ± 946.1	728.9 ± 123.7	751.1 ± 432.1
nab-PTX	83.2 ± 17	5.1 ± 1.0	36.5 ± 20.2	31.9 ± 8.6	1178.8 ± 301.2	441.4 ± 261.9	700.9 ± 200.6
micellar-PTX	43.8 ± 22	2.8 ± 1.5	15.73 ± 7.1	16.11 ± 8.0	719.1 ± 302.6	304.4 ± 172.4	335.8 ± 178.2

Abbreviation: br – brain, SC – spinal cord, SM – skeletal muscle, SN – sciatic nerve, DRG – dorsal root ganglia.

Table S6. Estimated steady-state total and unbound concentrations of paclitaxel in the brain (Br), spinal cord (SC), skeletal muscle (SM), dorsal root ganglia (DRG) and sciatic nerve (SN) with total drug concentrations in the respective tissues indicated. $f_{u,plasma}$, $K_{p,tissue}$ and $K_{p,uu,tissue}$ measured in the current study were used for the calculations, assuming the same total or unbound plasma concentration for each paclitaxel formulation. The density between different tissues is assumed to be the same.

Paclitaxel formulation	$C_{tot,plasma}$ (ng/mL)	$C_{u,plasma}$ (ng/mL)	$C_{tot,br}$ (ng/mL)	$C_{tot,SC}$ (ng/mL)	$C_{tot,SM}$ (ng/mL)	$C_{tot,SN}$ (ng/mL)	$C_{tot,DRG}$ (ng/mL)	$C_{u,br}$ (ng/mL)	$C_{u,SC}$ (ng/mL)	$C_{u,SM}$ (ng/mL)	$C_{u,SN}$ (ng/mL)	$C_{u,DRG}$ (ng/mL)
Assumed to be the same total paclitaxel plasma concentration of 100 ng/mL												
CreEL-PTX	100	3.9	16	11	536	224	210	0.03	0.02	6.55	3.47	1.06
nab-PTX	100	6.1	44	39	1476	547	861	0.06	0.06	18.18	8.60	4.33
micellar-PTX	100	6.5	38	41	1754	712	783	0.05	0.06	21.45	11.12	3.90
Assumed to be the same unbound paclitaxel plasma concentration of 10 ng/mL												
CreEL-PTX	256.4	10	41	28	1374	574	538	0.06	0.04	16.80	8.90	2.70
nab-PTX	163.9	10	72	64	2419	897	1411	0.10	0.09	29.80	14.10	7.10
micellar-PTX	153.8	10	58	63	2698	1095	1203	0.08	0.09	33.00	17.10	6.00
